# Germline *BRCA* mutation and outcome in young-onset breast cancer (POSH): a prospective cohort study

**DOI:** 10.1016/S1470-2045(17)30891-4

**Published:** 2018-02

**Authors:** Ellen R Copson, Tom C Maishman, Will J Tapper, Ramsey I Cutress, Stephanie Greville-Heygate, Douglas G Altman, Bryony Eccles, Sue Gerty, Lorraine T Durcan, Louise Jones, D Gareth Evans, Alastair M Thompson, Paul Pharoah, Douglas F Easton, Alison M Dunning, Andrew Hanby, Sunil Lakhani, Ros Eeles, Fiona J Gilbert, Hisham Hamed, Shirley Hodgson, Peter Simmonds, Louise Stanton, Diana M Eccles

**Affiliations:** a Cancer Sciences Academic Unit, Faculty of Medicine, University of Southampton, Southampton, UK; b Southampton Clinical Trials Unit, University of Southampton, Southampton, UK; c Genetic Epidemiology and Genomic Informatics Group, Human Genetics, Faculty of Medicine, University of Southampton, Southampton, UK; d University Hospital Southampton NHS Foundation Trust, Southampton, UK; e Centre for Statistics in Medicine, Nuffield Department of Orthopaedics, Rheumatology & Musculoskeletal Sciences, University of Oxford, Oxford, UK; f Tumour Biology Department, Institute of Cancer, Barts and The London School of Medicine & Dentistry, London, UK; g Centre for Genomic Medicine, Division of Evolution and Genomic Sciences, University of Manchester MAHSC, St Mary's Hospital, Manchester, UK; h University of Texas MD Anderson Cancer Center, Houston, TX, USA; i Centre for Cancer Genetic Epidemiology, Department of Public Health and Primary Care, University of Cambridge, Cambridge, UK; j Department of Pathology, University of Leeds, Faculty of Medicine, Leeds, UK; k Discipline of Molecular & Cellular Pathology, Faculty of Medicine, University of Queensland, The Royal Brisbane & Women's Hospital, Brisbane, QLD, Australia; l Institute of Cancer Research, London, UK; m Department of Radiology, University of Cambridge, Cambridge Biomedical Campus, Cambridge, UK; n Guy's & St Thomas' Hospital, London, UK; o St George's Hospital, University of London, London, UK

## Abstract

**Background:**

Retrospective studies provide conflicting interpretations of the effect of inherited genetic factors on the prognosis of patients with breast cancer. The primary aim of this study was to determine the effect of a germline *BRCA1* or *BRCA2* mutation on breast cancer outcomes in patients with young-onset breast cancer.

**Methods:**

We did a prospective cohort study of female patients recruited from 127 hospitals in the UK aged 40 years or younger at first diagnosis (by histological confirmation) of invasive breast cancer. Patients with a previous invasive malignancy (except non-melanomatous skin cancer) were excluded. Patients were identified within 12 months of initial diagnosis. *BRCA1* and *BRCA2* mutations were identified using blood DNA collected at recruitment. Clinicopathological data, and data regarding treatment and long-term outcomes, including date and site of disease recurrence, were collected from routine medical records at 6 months, 12 months, and then annually until death or loss to follow-up. The primary outcome was overall survival for all *BRCA1* or *BRCA2* mutation carriers (*BRCA*-positive) versus all non-carriers (*BRCA*-negative) at 2 years, 5 years, and 10 years after diagnosis. A prespecified subgroup analysis of overall survival was done in patients with triple-negative breast cancer. Recruitment was completed in 2008, and long-term follow-up is continuing.

**Findings:**

Between Jan 24, 2000, and Jan 24, 2008, we recruited 2733 women. Genotyping detected a pathogenic *BRCA* mutation in 338 (12%) patients (201 with *BRCA1*, 137 with *BRCA2*). After a median follow-up of 8·2 years (IQR 6·0–9·9), 651 (96%) of 678 deaths were due to breast cancer. There was no significant difference in overall survival between *BRCA*-positive and *BRCA*-negative patients in multivariable analyses at any timepoint (at 2 years: 97·0% [95% CI 94·5–98·4] *vs* 96·6% [95·8–97·3]; at 5 years: 83·8% [79·3–87·5] *vs* 85·0% [83·5–86·4]; at 10 years: 73·4% [67·4–78·5] *vs* 70·1% [67·7–72·3]; hazard ratio [HR] 0·96 [95% CI 0·76–1·22]; p=0·76). Of 558 patients with triple-negative breast cancer, *BRCA* mutation carriers had better overall survival than non-carriers at 2 years (95% [95% CI 89–97] *vs* 91% [88–94]; HR 0·59 [95% CI 0·35–0·99]; p=0·047) but not 5 years (81% [73–87] *vs* 74% [70–78]; HR 1·13 [0·70–1·84]; p=0·62) or 10 years (72% [62–80] *vs* 69% [63–74]; HR 2·12 [0·82–5·49]; p= 0·12).

**Interpretation:**

Patients with young-onset breast cancer who carry a *BRCA* mutation have similar survival as non-carriers. However, *BRCA* mutation carriers with triple-negative breast cancer might have a survival advantage during the first few years after diagnosis compared with non-carriers. Decisions about timing of additional surgery aimed at reducing future second primary-cancer risks should take into account patient prognosis associated with the first malignancy and patient preferences.

**Funding:**

Cancer Research UK, the UK National Cancer Research Network, the Wessex Cancer Trust, Breast Cancer Now, and the PPP Healthcare Medical Trust Grant.

## Introduction

Although only 5% of breast cancers are diagnosed in women aged younger than 40 years, a high proportion of deaths from breast cancer occur in this age group, which includes a higher number of patients who carry a pathogenic *BRCA1* or *BRCA2* mutation compared with patients with onset of breast cancer at an older age.^1^, ^2^, ^3^ Second primary breast cancers are more frequent in high-risk gene carriers, and this higher frequency drives early genetic testing to inform surgical decision making; however, whether a germline *BRCA1* or *BRCA2* mutation has independent prognostic implications after an initial cancer diagnosis is unclear.

*BRCA1* loss of function mutations are associated with high-histological-grade, oestrogen-receptor-negative, progesterone-receptor-negative, and HER2-negative (triple negative) breast cancer with a basal-like gene expression profile.^4^ *BRCA*2-associated breast tumours are usually high-grade, oestrogen-receptor positive, and HER2-negative.^5^, ^6^ *BRCA1* mutation carriers have been reported to have enhanced sensitivity to neoadjuvant chemotherapy with cytotoxic drugs.^7^

**Research in context**

**Evidence before this study**

At the initiation of this cohort study (Dec 3, 1999), we searched the PubMed database using the search terms [*BRCA1* OR *BRCA2*] AND [breast cancer or breast neoplasm] AND [survival OR prognosis OR mortality] and identified a few published retrospective studies reporting prognosis in *BRCA* mutation carriers. On Dec 5, 2016, we did another PubMed search for studies of patients who carried a *BRCA1* or *BRCA2* mutation and their prognosis, using the following search terms: “(*BRCA*) AND (survival or prognosis or outcome or mortality) AND (breast neoplasms or breast neoplasm or breast cancer or breast tumour)”. Our search was not limited by date or language. We also hand-searched references cited in review papers for additional papers. Previous studies and meta-analyses have reported inconsistent effects of *BRCA1* and *BRCA2* mutations on the outcomes of early breast cancer with better, worse, and similar outcomes for patients with a *BRCA1* or *BRCA2* mutation compared with patients with sporadic breast cancer. These conflicting results might be explained by methodological issues with ascertainment biases introduced by retrospective and selective identification of cases, incomplete genetic testing, small numbers, an absence of adjustment for clinical variables, including treatment, and short follow-up.

**Added value of this study**

POSH is, to our knowledge, the largest prospective cohort study to compare breast cancer outcomes of patients with a *BRCA1* or *BRCA2* mutation with patients with sporadic cancer. Our findings showed that patients with young-onset breast cancer who have a *BRCA* mutation have a similar overall survival to non-carriers. However, in patients with triple-negative breast cancer, *BRCA* mutation carriers might have a survival advantage compared with non-carriers during the first few years after diagnosis. Our study was strengthened by unbiased recruitment, universal and central genetic testing at the end of the study, and comprehensive pathological, clinical, and follow-up data.

**Implications of all the available evidence**

Decisions about timing of risk-reducing surgery should take into account primary tumour prognosis and patient preference.

Published studies and meta-analyses have reported better, worse, and similar outcomes for patients with a *BRCA1* or *BRCA2* mutation compared with patients with sporadic breast cancer.^8^, ^9^, ^10^, ^11^, ^12^, ^13^, ^14^ A comprehensive meta-analysis of 66 studies of breast cancer survival in patients with a *BRCA1* or *BRCA2* mutation compared with non-carrier patients or the general breast cancer population, which assessed study quality as well as outcome data, concluded that “it is not yet possible to draw evidence based conclusions about the association between *BRCA1* [or] *BRCA2* mutation carriership and breast cancer prognosis”.^12^ We undertook the Prospective Outcomes in Sporadic versus Hereditary breast cancer (POSH) study, the primary aim of which was to determine the effect of inherited *BRCA1* or *BRCA2* mutations on outcomes in patients with young-onset breast cancer.^15^, ^16^

## Methods

### Study design and participants

We did a prospective cohort study at 127 hospitals in the UK (appendix pp 1–2). We recruited young women (aged 18–40 years) diagnosed with primary breast cancer in the UK. Patients were eligible if they were diagnosed with invasive breast cancer aged 40 years or younger. Potential recruits were identified by local breast cancer clinicians, nurses, or research clinical trial practitioners within 12 months of initial diagnosis of invasive breast cancer and the date of diagnosis was defined as the first histological confirmation of invasive breast cancer. All histological subtypes, disease stages (I–IV), comorbidities, and performance statuses were permitted. Patients with a previous invasive malignancy (with the exception of non-melanomatous skin cancer) were excluded.

Written informed consent was obtained from all participants. Ethical approval was granted in 2000 (MREC 00/6/69) and the study was approved for recruitment as part of the UK National Cancer Research Network (NCRN) portfolio in 2002, subsequently the NIHR portfolio. The protocol was published in 2007.^15^

### Procedures

All patients received treatment according to local protocols. Details of personal characteristics, tumour pathology, disease stage, and surgical and cytotoxic treatment data were collected from medical records at study entry. Family history was collected by questionnaire. The BOADICEA algorithm, without adjustment for pathological subtype, was used to estimate the probability that an individual might carry a *BRCA1* or *BRCA2* pathogenic variant.^17^ Pathology and imaging data were verified with copies of the original reports from sites. For patients treated with neoadjuvant chemotherapy, the initial diameter of the tumour was derived from radiological reports.

The oestrogen-receptor, progesterone-receptor, and HER2-receptor status of the primary tumours was determined from reports of local routine pathology testing of diagnostic core biopsies or tumour resections for clinical use. Hormone-receptor concentrations equivalent to an Allred score of 3 or more were categorised as positive. Immunohistochemical staining of tissue microarrays in some cases enabled clinical source data for oestrogen-receptor, progesterone-receptor, and HER2-receptor statuses to be corroborated; tissue microarray scores were used to supplement missing datapoints for these receptors.^16^

DNA for genotyping was extracted from whole blood samples submitted at recruitment. A multiplex amplicon-based library preparation system, Fluidigm Access Array (Fluidigm UK, Cambridge, UK), targeted a panel of breast-cancer-susceptibility genes (including *BRCA1, BRCA2*, and *TP53*) for sequencing using an Illumina HiSeq2500 Next Generation Sequencing Platform (Illumina, Little Chesterford, UK; appendix pp 20–21). Targeted-sequence capture cannot reliably identify large exonic deletions or duplications, therefore multiplex ligation probe analysis was used for patients who met current UK guideline thresholds for clinical genetic testing.^17^, ^18^ Predicted protein truncating variants (frameshift, nonsense, and canonical-splice site and large rearrangements) plus other variants (mainly mis-sense) unequivocally defined as pathogenic on the basis of multiple lines of evidence and expert review were assigned to the *BRCA*-mutation carrier group (*BRCA*-positive). All pathogenic variants were confirmed by Sanger sequencing. All other patients, including those with *BRCA1* or *BRCA2* variants of uncertain significance or very low penetrance, were assigned to the same group as no mutation found (*BRCA*-negative) or excluded if they were found to carry a pathogenic variant of *TP53*. For the purposes of this analysis, mutations in other breast cancer genes were not curated.

The study protocol and patient information specified that patients would not be informed of the research genetic-testing results; however, patient information sheets gave information about seeking clinical genetic referral. Clinical referrals for genetic testing were made by the treating physician according to local protocols. Genetic test reports for the study patients generated by UK National Health Service (NHS) diagnostic laboratories were collected as part of the medical record.

Detailed clinical follow-up data, including date and site of disease recurrence, were obtained from medical records at 6 months, 12 months, and annually thereafter, until death or loss to follow-up. Patients were flagged in the NHS medical research information service for automatic notification of date and cause of death.

### Outcomes

The primary outcome was overall survival, defined as the time from first diagnosis to death from any cause. The secondary outcomes were distant disease-free survival, defined as time from first diagnosis to first distant disease excluding local (in breast) recurrence.

### Statistical analysis

The original study sample size of a minimum of 2000 patients was estimated based on a prevalence of *BRCA1* or *BRCA2* pathogenic mutations of 10%, and an absolute difference in event rate at 2 years between mutation carriers and non-carriers of 10% (20% in mutation carriers compared with 10% in sporadic cases).^15^ We also considered a prevalence of *BRCA1* or *BRCA2* mutations of 5% and 15%, and larger sample sizes. Good recruitment and data returns enabled us to continue study recruitment beyond 2000 participants providing sufficient power for multivariable analyses.

We did the statistical analyses according to a prespecified plan (appendix pp 22–31).^19^ The analysis population included all eligible patients recruited to the cohort who had available data for the primary tumour and genotyping, were aged 40 years or younger at the date of diagnosis, did not carry a *TP53* gene, and who did not present with metastatic disease at presentation (M1 stage). A prespecified subgroup of the analysis population was patients with triple-negative breast cancer (ie, oestrogen-receptor-negative, HER2-negative, and progesterone-receptor-negative or unknown). All analyses were done for both the overall analysis population and the triple-negative breast cancer subgroup population, unless specified otherwise. Key patient data were described by *BRCA* mutation status, and formal comparisons by *BRCA* mutation status were done using Mann-Whitney tests (for continuous variables) and Pearson χ^2^ tests (for categorical variables) for patients with complete data. We used Kaplan-Meier plots to show survival data by *BRCA* status at 2, 5, and 10 years. The 2-year comparison was chosen because this timepoint was specified for the original sample size; the 5-year and 10-year comparisons were chosen because they are commonly used in such studies and are clinically relevant timepoints. Patients who did not have an event were censored at the date of their last follow-up. Hazard ratios (HRs) and 95% CIs for univariable analyses and multivariable analyses (for the primary and secondary outcomes) were calculated using Cox proportional-hazards models, or flexible parametric survival models for those that involved time-varying hazards.^20^ For each flexible parametric survival model, varying degrees of freedom for the baseline-hazard rate and time-dependent effect were explored to obtain the best-model fit. All missing data were assumed to be either missing at random or missing completely at random, and censoring was assumed to be non-informative. Prespecified sensitivity analyses included the generation of corresponding complete-case multivariable analysis model results.

Post-hoc sensitivity analyses were done to explore the possible reasons for some of the results in the triple-negative breast cancer group. Additionally, to investigate the degree of potential bias from time of diagnosis to blood draw for genetic testing at registration, a multivariable analysis model adjusting for the time from diagnosis to blood draw was generated accordingly for the analysis population only. We considered if the longer survival of *BRCA* mutation carriers with triple-negative breast cancer could be due to a beneficial effect of risk-reducing surgery in *BRCA* carriers, so we repeated the analysis in this subgroup excluding patients who underwent bilateral mastectomy within the first year after diagnosis. A further sensitivity analysis was done to compare the pattern of improved survival at an early timepoint with apparently worse survival in the long term by excluding patients who developed a new primary breast or ovarian cancer.

We did all analyses with Stata, version 14.2, and multiple imputation was incorporated in the multivariable analyses generated using the mi command.

### Role of the funding source

The funders and their representatives had no role in study design, data collection, data analysis, data interpretation, or writing of the report or the decision to submit it for publication. The corresponding author had full access to all the data in the study and had final responsibility for the decision to submit for publication.

## Results

**Figure 1:**
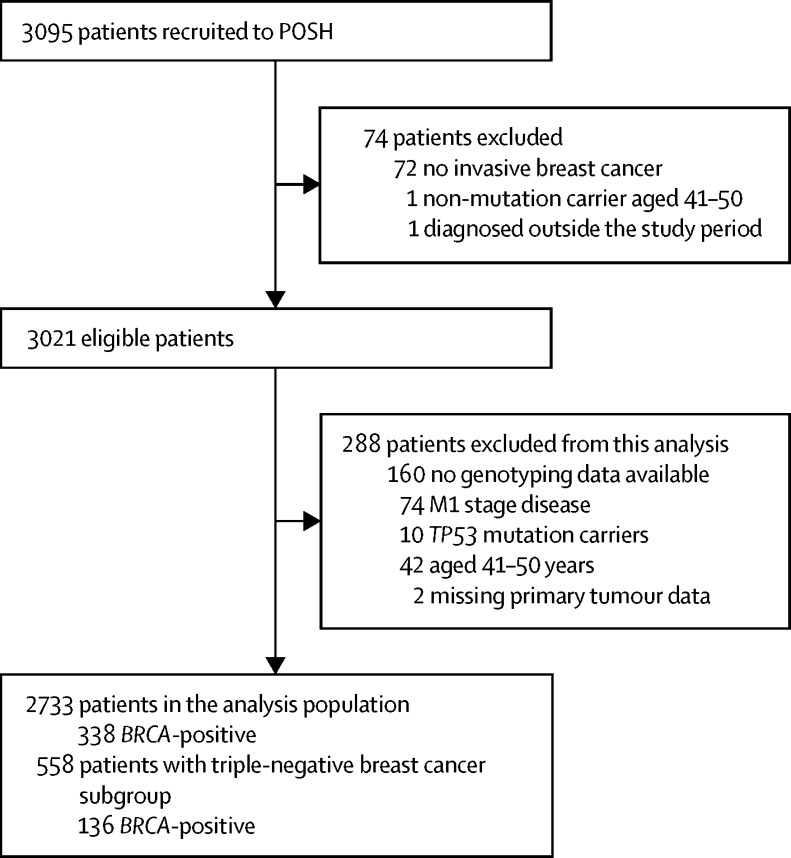
Trial profile *BRCA*-positive=patient with *BRCA1* or *BRCA2* pathogenic mutation. Patients were categorised as *BRCA*-negative if no *BRCA* pathogenic mutation was found or they had a *BRCA1* or *BRCA2* variant of uncertain significance or very low penetrance.

Between Jan 24, 2000, and Jan 24, 2008, we recruited 3021 eligible women, of whom 2733 (91%) were included in the analysis population, and 288 (9%) were excluded (figure 1; appendix p 11). We included all data received until July 26, 2016. Of 2721 patients for whom presentation was recorded, 45 (2%) were recorded as being enrolled in a surveillance programme, and 33 (1%) were recorded as having screen-detected breast cancer. Screening was offered according to local protocols; national guidelines were not formally established until after recruitment ended.

338 (12%) of 2733 patients included in the analysis population had either a *BRCA1* or *BRCA2* mutation, of whom 44 (13%) had large-copy-number variants (appendix pp 3–7). 75 (22%) of 338 patients did not meet current family history or pathology based genetic-testing guidelines.^18^ Referral for a clinical genetics consultation and *BRCA* testing occurred for 388 patients (14%), of whom 182 (47%) had a pathogenic mutation. Immunohistochemical staining of tissue microarrays in 1336 cases, during 2012 and 2016, enabled clinical source data for oestrogen-receptor, progesterone-receptor, and HER2-receptor statuses to be corroborated.

The median time from breast cancer diagnosis to study registration blood draw was 5·5 months (IQR 3·2–10·7). There were several significant clinicopathological differences between *BRCA*-positive and *BRCA*-negative patients, and between *BRCA1* mutation carriers and *BRCA2* mutation carriers (table 1). The most commonly used chemotherapy regimen was anthracycline with or without taxanes. Of the 2733 patients in the analysis population, 558 (20%) had triple-negative breast cancer. *BRCA* mutations were identified in 136 (24%) of patients with triple-negative breast cancer, of whom 123 (90%) had a *BRCA1* mutation. Differences in tumour characteristics between *BRCA1* and *BRCA2* mutation carriers were also noted in patients with triple-negative breast cancer (table 2).

**Table 1:** Baseline characteristics and clinicopathological information for all patients

		**All patients (n=2733)**	***BRCA1*-positive (n=201)**	***BRCA2*-positive (n=137)**	***BRCA*-positive (n=338)**	***BRCA*-negative (n=2395)**	p value*
Age at diagnosis (years)		36 (34–38, 18–40)	35 (32–38, 22–40)	37 (33–38, 21–40)	36 (32–38, 21–40)	37 (34–39, 18–40)	*BRCA*-positive *vs BRCA*-negative p<0·0001, *BRCA1*-positive *vs BRCA2*-positive p=0·014
BMI (kg/m^2^)							*BRCA*-positive *vs BRCA*-negative p=0·48, *BRCA1*-positive *vs BRCA2*-positive p=0·40
	<25	1427/2632 (54%)	114/192 (59%)	70/133 (53%)	184/325 (57%)	1243/2307 (54%)	
	≥25 to <30	714/2632 (27%)	47/192 (25%)	41/133 (31%)	88/325 (27%)	626/2307 (27%)	
	≥30	491/2632 (19%)	31/192 (16%)	22/133 (17%)	53/325 (16%)	438/2307 (19%)	
	Missing	101 (4%)	9 (5%)	4 (3%)	13 (4%)	88 (4%)	
Ethnicity							*BRCA*-positive *vs BRCA*-negative p=0·28, *BRCA1*-positive *vs BRCA2*-positive p=0·99
	White	2494/2698 (92%)	178/196 (91%)	122/134 (91%)	300/330 (91%)	2194/2368 (93%)	
	Black	103/2698 (4%)	10/196 (5%)	6/134 (5%)	16/330 (5%)	87/2368 (4%)	
	Asian	80/2698 (3%)	5/196 (3%)	4/134 (3%)	9/330 (3%)	71/2368 (3%)	
	Other	21/2698 (<1%)	3/196 (2%)	2/134 (2%)	5/330 (2%)	16/2368 (<1%)	
	Missing	35 (1%)	5 (3%)	3 (2%)	8 (2%)	27 (1%)	
Histological grade							*BRCA*-positive *vs BRCA*-negative p<0·0001, *BRCA1*-positive *vs BRCA2*-positive p<0·0001
	1	156/2658 (6%)	2/197 (1%)	0	2/326 (<1%)	154/2332 (7%)	
	2	904/2658 (34%)	16/197 (8%)	40/129 (31%)	56/326 (17%)	848/2332 (36%)	
	3	1598/2658 (60%)	179/197 (91%)	89/129 (69%)	268/326 (82%)	1330/2332 (57%)	
	Missing or not graded	75 (3%)	4 (2%)	8 (6%)	12 (4%)	63 (3%)	
Oestrogen-receptor status							*BRCA*-positive *vs BRCA*-negative p<0·0001, *BRCA1*-positive *vs BRCA2*-positive p<0·0001
	Negative	908/2719 (33%)	151/200 (76%)	21/136 (15%)	172/336 (51%)	736/2383 (31%)	
	Positive	1811/2719 (67%)	49/200 (25%)	115/136 (85%)	164/336 (49%)	1647/2383 (69%)	
	Missing	14 (<1%)	1 (<1%)	1 (<1%)	2 (<1%)	12 (<1%)	
*HER2* status							*BRCA*-positive *vs BRCA*-negative p<0·0001, *BRCA1*-positive *vs BRCA2*-positive p=0·18
	Negative	1763/2412 (73%)	164/176 (93%)	111/125 (89%)	275/301 (91%)	1488/2111 (71%)	
	Positive	649/2412 (27%)	12/176 (7%)	14/125 (11%)	26/301 (9%)	623/2111 (30%)	
	Missing	321 (12%)	25 (12%)	12 (9%)	37 (11%)	284 (12%)	
Progesterone-receptor status							*BRCA*-positive *vs BRCA*-negative p<0·0001, *BRCA1*-positive *vs BRCA2*-positive p<0·0001
	Negative	951/2208 (43%)	144/171 (84%)	23/107 (22%)	167/278 (60%)	784/1930 (41%)	
	Positive	1257/2208 (57%)	27/171 (16%)	84/107 (79%)	111/278 (40%)	1146/1930 (59%)	
	Missing	525 (19%)	30 (15%)	30 (22%)	60 (18%)	465 (19%)	
†Triple-negative breast cancer status							*BRCA*-positive *vs BRCA*-negative p<0·0001, *BRCA1*-positive *vs BRCA2*-positive p<0·0001
	No	2175/2733 (80%)	78/201 (39%)	124/137 (91%)	202/338 (60%)	1973/2395 (82%)	
	Yes	558/2733 (20%)	123/201 (61%)	13/137 (10%)	136/338 (40%)	422/2395 (18%)	
Maximum invasive tumour size (mm)		22 (15–33, 0–170)	21 (15–30, 1–140)	25 (16–32, 1–92)	22 (15–31, 1–140)	22 (15–34, 0–170)	*BRCA*-positive *vs BRCA*-negative p=0·97, *BRCA1*-positive *vs BRCA2*-positive p=0·060
	Missing	156 (6%)	10 (5%)	14 (10%)	24 (7%)	132 (6%)	
Pathological N stage							*BRCA*-positive *vs BRCA*-negative p=0·013, *BRCA1*-positive *vs BRCA2*-positive p<0·0001
	0	1304/2692 (48%)	129/201 (64%)	55/135 (41%)	184/336 (55%)	1120/2356 (48%)	
	1	1388/2692 (52%)	72/201 (36%)	80/135 (59%)	152/336 (45%)	1236/2356 (53%)	
Axillary nodal involvement							*BRCA*-positive *vs BRCA*-negative p=0·019, *BRCA1*-positive *vs BRCA2*-positive p=0·00017
	1–3	899/2692 (33%)	43/201 (21%)	51/135 (38%)	94/336 (28%)	805/2356 (34%)	
	4–9	330/2692 (12%)	14/201 (7%)	19/135 (14%)	33/336 (10%)	297/2356 (13%)	
	≥10	159/2692 (6%)	15/201 (8%)	10/135 (7%)	25/336 (7%)	134/2356 (6%)	
	Missing	41 (2%)	0	2 (2%)	2 (<1%)	39 (2%)	
Lymphovascular invasion							*BRCA*-positive *vs BRCA*-negative p=0·23, *BRCA1*-positive *vs BRCA2*-positive p=0·013
	Absent	1327/2539 (52%)	116/190 (61%)	58/124 (47%)	174/314 (55%)	1153/2225 (52%)	
	Present	1212/2539 (48%)	74/190 (39%)	66/124 (53%)	140/314 (45%)	1072/2225 (48%)	
	Missing	194 (7%)	11 (6%)	13 (10%)	24 (7%)	170 (7%)	
Chemotherapy							*BRCA*-positive *vs BRCA*-negative p=0·0058, *BRCA1*-positive *vs BRCA2*-positive p=0·016
	None	294/2733 (11%)	9/201 (5%)	11/137 (8%)	20/338 (6%)	274/2395 (11%)	
	Adjuvant	2027/2733 (74%)	171/201 (85%)	99/137 (72%)	270/338 (80%)	1757/2395 (73%)	
	Neoadjuvant	412//2733 (15%)	21/201 (10%)	27/137 (20%)	48/338 (14%)	364/2395 (15%)	
Type of surgery							*BRCA*-positive *vs BRCA*-negative p=0·30, *BRCA1*-positive *vs BRCA2*-positive p=0·00040
	Breast-conserving surgery	1337/2733 (49%)	106/201 (53%)	43/137 (31%)	149/338 (44%)	1188 (50%)	
	Mastectomy	1373/2733 (50%)	94/201 (47%)	92/137 (67%)	186/338 (55%)	1187/2395 (50%)	
	Nodal surgery only	7/2733 (<1%)	1/201 (<1%)	0	1/338 (<1%)	6/2395 (<1%)	
	None	16/2733 (<1%)	0	2/137 (2%)	2/338 (<1%)	14/2395 (<1%)	
Chemotherapy regimen							*BRCA*-positive *vs BRCA*-negative p=0·015, *BRCA1*-positive *vs BRCA2*-positive p=0·38
	None	294/2733 (11%)	9/201 (5%)	11/137 (8%)	20/338 (6%)	274/2395 (11%)	
	Anthracyclines	1760/2733 (64%)	145/201 (72%)	89/137 (65%)	234/338 (69%)	1526/2395 (64%)	
	Taxanes	24/2733 (<1%)	0	1/137 (<1%)	1/338 (<1%)	23/2395 (1%)	
	Anthracyclines and taxanes	635/2733 (23%)	45/201 (22%)	34/137 (25%)	79/338 (23%)	556/2395 (23%)	
	Other (including CMF)	20/2733 (<1%)	2/201 (1%)	2/137 (2%)	4/338 (1%)	16/2395 (<1%)	

Data are median (IQR, range) or n (%). Patients with missing data were not included in the p value calculation. BMI=body-mass index. CMF=cyclophosphamide plus methotrexate plus fluorouracil.

* Test excluded patients with both *BRCA1* and *BRCA2* mutations. Mann-Whitney tests used for continuous variables and Pearson χ^2^ tests for categorical variables, done on patients with complete data.

† Defined as oestrogen-receptor-negative, HER2-negative, and progesterone-receptor-negative or unknown.

**Table 2:** Baseline characteristics and clinicopathological information for patients with triple-negative breast cancer*

		**All patients (n=558)**	***BRCA1*-positive (n=123)**	***BRCA2*-positive (n=13)**	***BRCA*-positive (n=136)**	***BRCA*-negative (n=422)**	**p value**†
Age at diagnosis (years)		36 (33–38, 19–40)	34 (32–37, 22–40)	33 (32–38, 30–40)	34 (32–37, 22–40)	36 (33–38, 19–40)	*BRCA*-positive *vs BRCA*-negative p=0·00056, *BRCA1*-positive *vs BRCA2*-positive p=0·79
BMI (kg/m^2^)							*BRCA*-positive *vs BRCA*-negative p=0·26, *BRCA1*-positive *vs BRCA2*-positive p=0·47
	<25	274/546 (50%)	67/119 (56%)	5/13 (39%)	72/132 (55%)	202/414 (49%)	
	≥25 to <30	149/546 (27%)	32/119 (27%)	5/13 (39%)	37/132 (28%)	112/414 (27%)	
	≥30	123/546 (23%)	20/119 (17%)	3/13 (23%)	23/132 (18%)	100/414 (24%)	
	Missing	12 (2%)	4 (3%)	0	4 (3%)	8 (2%)	
Ethnicity							*BRCA*-positive *vs BRCA*-negative p=0·52, *BRCA1*-positive *vs BRCA2*-positive p=0·052
	White	500/550 (91%)	110/122 (90%)	9/13 (69%)	119/135 (88%)	381/415 (92%)	
	Black	26/550 (5%)	7/122 (6%)	2/13 (15%)	9/135 (7%)	17/415 (4%)	
	Asian	19/550 (4%)	3/122 (3%)	2/13 (15%)	5/135 (4%)	14/415 (3%)	
	Other	5/550 (<1%)	2/122 (2%)	0	2/135 (2%)	3/415 (<1%)	
	Missing	8 (1%)	1 (<1%)	0	1 (<1%)	7 (2%)	
Histological grade							*BRCA*-positive *vs BRCA*-negative p=0·49, *BRCA1*-positive *vs BRCA2*-positive p=0·41
	1	3/541 (<1%)	0	0	0	3/406 (<1%)	
	2	30/541 (6%)	6/122 (5%)	0	6/135 (4%)	24/406 (6%)	
	3	508/541 (94%)	116/122 (95%)	13/13 (100%)	129/135 (96%)	379/406 (93%)	
	Missing or not graded	17 (3%)	1 (<1%)	0	1 (<1%)	16 (4%)	
Maximum invasive tumour size (mm)		22 (15–31, 1–160)	21 (15–30, 4–140)	23 (16–30, 15–30)	21 (15–30, 4–140)	23 (15–32, 1–160)	*BRCA*-positive *vs BRCA*-negative p=0·17, *BRCA1*-positive *vs BRCA2*-positive p=0·72
	Missing	35 (6%)	5 (4%)	3 (23%)	8 (6%)	27 (6%)	··
Pathological N stage							*BRCA*-positive *vs BRCA*-negative p=0·46, *BRCA1*-positive *vs BRCA2*-positive p=0·64
	0	341/552 (62%)	80/123 (65%)	7/12 (58%)	87/135 (64%)	254/417 (61%)	
	1	211/552 (38%)	43/123 (35%)	5/12 (42%)	48/135 (36%)	163/417 (39%)	
Axillary nodal involvement							*BRCA*-positive *vs BRCA*-negative p=0·044, *BRCA1*-positive *vs BRCA2*-positive p=0·68
	1 to 3	141/552 (26%)	26/123 (21%)	4/12 (33%)	30/135 (22%)	111/417 (27%)	
	4 to 9	45/552 (8%)	7/123 (6%)	0	7/135 (5%)	38/417 (9%)	
	≥10	25/552 (5%)	10/123 (8%)	1/12 (8%)	11/135 (8%)	14/417 (3%)	
	Missing	6 (1%)	0	1 (8%)	1 (<1%)	5 (1%)	
Lymphovascular invasion							*BRCA*-positive *vs BRCA*-negative p=0·83, *BRCA1*-positive *vs BRCA2*-positive p=0·19
	Absent	312/517 (60%)	71/116 (61%)	4/10 (40%)	75/126 (60%)	237/391 (61%)	
	Present	205/517 (40%)	45/116 (39%)	6/10 (60%)	51/126 (41%)	154/391 (39%)	
	Missing	41 (7%)	7 (6%)	3 (23%)	10 (7%)	31 (7%)	
Chemotherapy							*BRCA*-positive *vs BRCA*-negativep=0·17, *BRCA1*-positive *vs BRCA2*-positive, p=0·074
	None	13/558 (2%)	3/123 (2%)	0	3/136 (2%)	10/422 (2%)	
	Adjuvant	450/558 (81%)	108/123 (88%)	9/13 (69%)	117/136 (86%)	333/422 (79%)	
	Neoadjuvant	95/558 (17%)	12/123 (10%)	4/13 (31%)	16/136 (12%)	79/422 (19%)	
Type of surgery							*BRCA*-positive *vs BRCA*-negative p=0·19, *BRCA1*-positive *vs BRCA2*-positive p=0·014
	Breast-conserving surgery	331/558 (59%)	69/123 (56%)	5/13 (39%)	74/136 (54%)	257/422 (61%)	
	Mastectomy	223/558 (40%)	53/123 (43%)	7/13 (54%)	60/136 (44%)	163/422 (39%)	
	Nodal surgery only	1/558 (<1%)	1/123 (<1%)	0	1/136 (<1%)	0	
	None	3/558 (<1%)	0	1/13 (8%)	1/136 (<1%)	2/422 (<1%)	
Chemotherapy regimen							*BRCA*-positive *vs BRCA*-negative p=0·097, *BRCA1*-positive *vs BRCA2*-positive p=0·086
	None	13 (2%)	3 (2%)	0	3 (2%)	10 (2%)	
	Anthracyclines	382/558 (69%)	91/123 (74%)	6/13 (46%)	97/136 (71%)	285/422 (68%)	
	Taxanes	2/558 (<1%)	0	0	0	2/422 (<1%)	
	Anthracyclines and taxanes	159/558 (29%)	27/123 (22%)	7/13 (54%)	34/136 (25%)	125/422 (30%)	
	Other (includes CMF)	2/558 (<1%)	2/123 (2%)	0	2/136 (2%)	0	

Data are median (IQR, range) or n (%). Patients with missing data were not included in the p value calculation. BMI=body-mass index. CMF=cyclophosphamide plus methotrexate plus fluorouracil.

* Defined as oestrogen-receptor-negative, HER2-negative, and progesterone-receptor-negative or unknown.

† Test excluded patients with both *BRCA1* and *BRCA2* mutations. Mann-Whitney tests used for continuous variables and Pearson χ^2^-tests for categorical variables, done on patients with complete data.

Median follow-up was 8·2 years (IQR 6·0–9·9); 91 (3%) patients were lost to follow-up. Contralateral breast tumours occurred in 151 (6%) patients: in 37 (18%) of 201 *BRCA1* mutation carriers, 17 (12%) of 137 *BRCA*2 mutation carriers, and 97 (4%) of 2395 *BRCA*-negative patients. Median time to contralateral breast cancer was 3·0 years (IQR 1·5–4·8) in *BRCA*-positive patients and 2·7 years (1·2–5·3) in *BRCA*-negative patients. 752 (28%) women developed a distant recurrence. Of 678 deaths, 651 (96%) were due to breast cancer. Deaths due to non-breast malignancies included six (3%) of 201 new primary cancers in *BRCA1* mutation carriers (three ovarian, one primary peritoneal, one oesophageal, and one pancreatic) and 12 (<1%) of 2395 malignancies in *BRCA*-negative patients (four haematological, three lung, and one each of brain, colorectal, gastric, pancreatic, and sarcoma; appendix p 8). There were no deaths attributed to second primary cancers among *BRCA2* mutation carriers.

**Figure 2:**
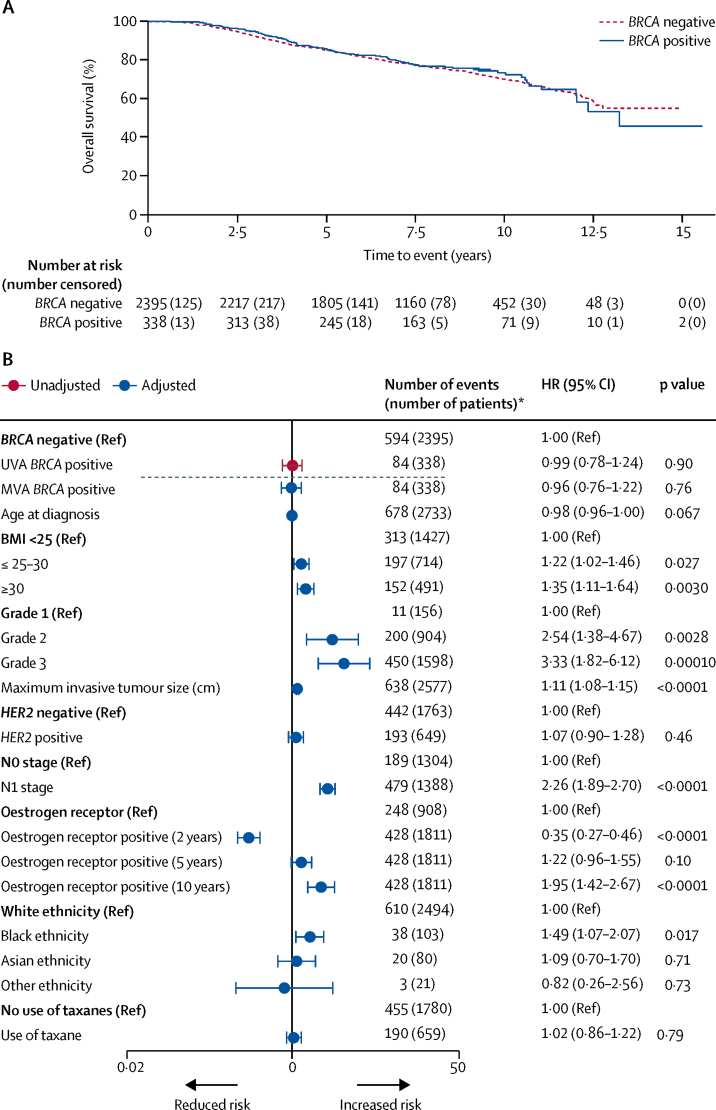
Overall survival for all patients (analysis population) by *BRCA* mutation status (A) Kaplan-Meier plot and (B) forest plot of corresponding univariable and multivariable hazard ratios. In (B), multivariable analysis was adjusted for age, body-mass index (BMI; kg/m^2^), grade, tumour size, HER2 status, oestrogen-receptor status, ethnicity, and use of taxane chemotherapy. Groups without a reference were assessed as a continuous variable. The dashed line separates the univariable analysis (UVA) from the multivariable analysis (MVA). Oestrogen-receptor-positive group assessed at 2, 5, and 10 years because the hazard ratio associated with oestrogen-positive status varies with time.^16^ HR=hazard ratio. *Number of events (number of patients) from complete data obtained before multiple imputation.

Overall survival was 97·0% (95% CI 94·5–98·4) in *BRCA*-positive patients versus 96·6% (95·8–97·3) in *BRCA*-negative patients at 2 years; 83·8% (79·3–87·5) versus 85·0% (83·5–86·4) at 5 years; and 73·4% (67·4–78·5) versus 70·1% (67·7–72·3) at 10 years (figure 2). There was no difference in overall survival between groups either before or after adjusting for known prognostic factors, including adjustments for ethnicity and body-mass index (BMI; univariable analysis negative *vs* positive HR 0·99 [95% CI 0·78–1·24], p=0·90; multivariable analysis HR 0·96 [0·76–1·22], p=0·76). Similar results were noted when comparing distant disease-free survival between *BRCA*-positive and *BRCA*-negative groups (appendix p 12). Additionally, comparison of overall survival in *BRCA*-negative patients versus *BRCA1* or *BRCA2* carriers separately showed similar results (appendix pp 13–14).

**Figure 3:**
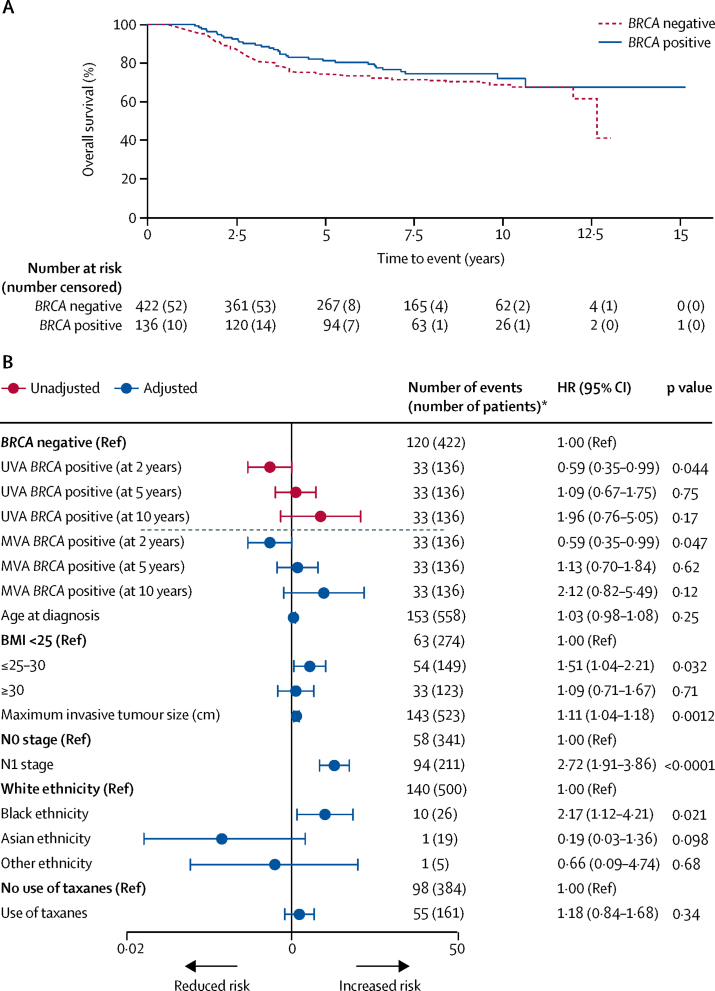
Overall survival for all patients with triple-negative breast cancer* by *BRCA* mutation status (A) Kaplan-Meier plot and (B) forest plot of corresponding univariable and multivariable hazard ratios. In (B), multivariable analysis was adjusted for age, body-mass index (BMI; kg/m^2^), grade, tumour size, HER2 status, oestrogen-receptor status, ethnicity, and use of taxane chemotherapy. Groups without a reference were assessed as a continuous variable. The dashed line separates the univariable analyses (UVA) from the multivariable analyses (MVA). HR=hazard ratio. *Number of events (number of patients) from complete data obtained before multiple imputation.

In the subgroup of 558 patients with triple-negative breast cancer, 159 (28%) women developed a distant recurrence, 153 (27%) died, and all deaths were due to breast cancer. The estimated hazard for death after diagnosis of triple-negative breast cancer varied over time (appendix p 32). In the triple-negative breast cancer subgroup, overall survival was significantly better at 2 years for *BRCA*-positive patients than for *BRCA*-negative patients (95% [95% CI 89–97]) *vs* 91% [88–94]; multivariable analysis flexible parametric survival model HR 0·59 [95% CI 0·35–0·99], p=0·047). Overall survival at 5 years was 81% (95% CI 73–87) versus 74% (70–78; multivariable analysis flexible parametric survival model HR 1·13 [95% CI 0·70–1·84], p=0·62); and at 10 years was 72% (62–80) versus 69% (63–74; multivariable analysis flexible parametric survival model HR 2·12 [95% CI 0·82–5·49], p=0·12; figure 3). For distant disease-free survival, however, the difference between *BRCA*-positive and *BRCA*-negative patients was not significant (appendix p 15). Inclusion of time from diagnosis to registration blood draw in multivariable analyses did not affect the results (appendix p 16). For analyses of both the overall population and the subgroup of patients with triple-negative breast cancer, results with imputation were almost identical to complete case results (appendix pp 9–10). Results from tests of proportional hazards are also in the appendix (p 17).

A post-hoc, multivariable sensitivity analysis of overall survival in patients with triple-negative breast cancer excluding 31 (6%) patients (21 *BRCA*-positive and ten *BRCA*-negative) who underwent bilateral mastectomy within the first year after diagnosis showed a significant difference in overall survival at 2 years for *BRCA*-positive versus *BRCA*-negative patients (95% [95% CI 89–98] *vs* 91% [88–94]; HR 0·52 [95% CI 0·29–0·91], p=0·023). However, there was no significant difference for 5-year overall survival (83% [95% CI 74–89] *vs* 74% [69–78]; HR 0·98 [95% CI 0·58–1·65], p=0·94; appendix p 18). We also repeated the primary analysis in patients with triple-negative breast cancer excluding 37 (7%) patients who developed a new primary breast or ovarian cancer. Overall survival at 10 years for *BRCA*-positive versus *BRCA-*negative patients was 78% (95% CI 69–85) versus 69% (64–74; HR 1·24 [95% CI 0·39–3·96], p=0·73; appendix p 19).

## Discussion

The POSH prospective cohort study showed no significant difference in overall survival or distant disease-free survival between patients carrying a *BRCA1* or *BRCA2* mutation and patients without these mutations after a diagnosis of breast cancer. These results did not vary between unadjusted or adjusted analyses, including adjustments for ethnicity and BMI.^21^, ^22^ Following a diagnosis of early breast cancer, *BRCA* mutation carriers are frequently offered additional management options including bilateral mastectomy. Any prognostic implication of carrying a *BRCA* mutation for primary treatment is important to clarify to facilitate clinician and patient decisions around the optimum timing of additional surgery. Furthermore, clinical trials of treatments that are specifically targeted toward *BRCA* mutation carriers might need to take into account any effect of *BRCA* mutational status on primary treatment outcomes.

To our knowledge, this is the largest prospective study to report the prognostic implication of germline *BRCA* mutations and the only one with a preplanned analysis of patients presenting with triple-negative tumours. Our results are in broad agreement with more recent studies,^8^, ^9^, ^10^, ^23^ but others have reported conflicting results.^24^, ^25^, ^26^ Ascertainment biases introduced by retrospective and selective identification of cases, incomplete genetic testing, small numbers, absence of adjustments for clinical variables including treatment, and short follow-up probably explain many discrepancies, although some studies have generally used stronger methods.^11^, ^12^, ^13^, ^14^

The percentage of *BRCA*-positive patients in POSH (12%) was higher than anticipated from historical studies of patients diagnosed aged 40 years and younger, perhaps because of more sensitive mutation-testing options.^1^ However, only 14% of all patients had clinical genetic testing. The ratio of patients with *BRCA1* to *BRCA*2 mutations was 1·5 to 1, which is similar to that reported in other large western population-based cohorts.^2^, ^23^ Deaths due to other malignancies were low in frequency in all groups reflecting the young age group; however, causes of deaths in patients who were *BRCA1*-positive included potentially preventable ovarian cancers at age 41–46 years. Bilateral risk-reducing mastectomy is not a necessary part of treating a unilateral breast cancer but unilateral mastectomy might enable breast radiotherapy to be omitted. Discussion about future primary cancer prevention during primary breast cancer treatment should take into account individual circumstances, including the likely tumour prognosis and the physical and psychological implications of more extensive surgery. In the POSH cohort, immediate bilateral mastectomy was not associated with improved survival, although the reported use of risk-reducing surgery was low; bilateral salpingo-oophorectomy was recorded in 32 patients and bilateral mastectomies in 107 patients.^27^ This probably reflects the low level of clinical testing at the time of the study. Although risk-reducing bilateral salpingo-oophorectomy is highly effective at reducing ovarian cancer incidence, the risk of primary peritoneal cancer is not reduced and studies indicate that the previously reported effect of this procedure on future breast cancer risk in *BRCA1* and *BRCA2* mutation carriers might have been overestimated because of uncorrected bias.^28^

Our analysis of the 558 patients with triple-negative breast cancer in our cohort showed an intriguing difference in overall survival over the first few years after diagnosis. *BRCA* mutation carriers were less likely to die from early breast cancer than non-carriers. This early survival advantage has also been observed among patients with ovarian cancer who are *BRCA* mutation carriers.^29^, ^30^ If real, this advantage might reflect greater sensitivity of *BRCA*-mutant breast cancers to chemotherapy or the greater visibility of *BRCA*-mutant cancers to host immune attack.^31^ One theory that could explain the slight survival advantage for *BRCA* mutation carriers not undergoing immediate bilateral mastectomy is that a major surgical intervention might compromise host immunity at a time when this is particularly important for eradicating micrometastases. This hypothesis would need further exploration due to the small number of patients in this subgroup.

Results from several published studies have suggested that the DNA repair deficiency associated with *BRCA* mutations results in enhanced sensitivity to many chemotherapy agents, particularly higher response rates to platinum-based drugs, have occurred in both metastatic and neoadjuvant settings.^4^, ^7^ Only 13 patients in our cohort were treated with platinum-based adjuvant regimens for early breast cancer, including one patient with a *BRCA1* mutation and one with *BRCA2*.

Our study illustrates the high breast cancer mortality in this unscreened young population and the effect of known tumour and patient-prognostic characteristics on mortality. Inevitably, there have been substantial changes in the management of *BRCA1* and *BRCA2* mutation carriers since the recruitment period of this study, including the exploration in trials of systemic therapies that exploit *BRCA-*null tumours, including platinum-based drugs and PARP inhibitors. The association of *BRCA* mutations with improved early outcomes related to breast cancer in patients with triple-negative breast cancer has the potential to affect early results from clinical trials. As advanced genomic investigations increasingly become a part of routine oncological care, many patients with breast cancer now learn their *BRCA* mutation status close to the time of diagnosis. In many cancer centres, immediate or post-chemotherapy bilateral mastectomy has become an almost routine recommendation for *BRCA1* and *BRCA2* mutation carriers regardless of the size or focality of the presenting tumour. In the longer term, risk-reducing surgery, particularly for *BRCA1* gene carriers is an appropriate management; in our analysis, the rising hazard for death in *BRCA* carriers over time was negated by removing from the analysis all patients who developed a second new primary breast or ovarian cancer during the follow-up period.

Clinicians need to consider short-term and long-term risks and benefits in discussing risk-reducing bilateral mastectomy with patients. The number of patients with triple-negative breast cancer who had immediate bilateral mastectomy in our cohort was small but our analysis suggests it is unlikely that the early bilateral mastectomy accounted for the early survival advantage in the *BRCA* mutation carriers with triple-negative breast cancer. With modern MRI-based breast screening, we conclude that patients who choose to delay additional surgery for 1 or 2 years until they are psychologically and physically recovered from their cancer treatment can be reassured that this choice is unlikely to lead to any substantial survival disadvantage. The importance of appropriately timed risk-reducing bilateral salpingo-oophorectomy, for *BRCA1* mutation carriers in particular, is clear, but should take plans for further pregnancy into account. Furthermore, risk-reducing bilateral salpingo-oophorectomy in very young women will have negative health consequences as a result of oestrogen deprivation from an early age.

The strengths of the POSH study include the large cohort size, few missing data, and inclusion of patients with young-onset breast cancer, which led to a large number of *BRCA1* and *BRCA2* mutation carriers and a high number of events, ensuring that the study was well powered for the main outcome analysis. Our study minimised many of the biases present in other studies by recruiting patients within the first year after diagnosis from oncology clinics nationally to minimise survival and selection bias and by establishing *BRCA* mutation status for all patients included in the analysis. POSH participants recruited from England represented 23% of the available population during the recruitment period and comparison with cancer registry data confirmed that the POSH cohort is representative of the wider population.^16^ Comprehensive details of pathology enabled us to do a separate analysis of outcome in patients with triple-negative breast tumours; a unique contribution to this field. We have previously reported the significant and independent prognostic effects of obesity and ethnicity on long-term outcomes in this young patient group, and this study is the only prospective study to date to include these host factors in multivariable analyses.^21^, ^22^

Limitations of this study included the non-universal use of multiplex ligation probe analysis; we therefore cannot exclude the possibility that some structural *BRCA* variants were not identified. However, even clinical diagnostic mutation testing is not 100% sensitive because of occult mutations not amenable to current methods (eg, deep intronic splice variants); the investigation of *BRCA1* and *BRCA2* gene sequences in this cohort was more comprehensive than in most other publications. All participants were tested for *TP53* mutations and carriers were excluded from this analysis because of the high risk of non-breast malignancies. We acknowledge that other breast cancer susceptibility gene variants were not excluded; however, these were expected to be very low in frequency or low penetrance, and there is no evidence that they specifically affect prognosis. We had national outcome data up to a median 8·2 years. The treatments given reflected modern oncological practice with almost 90% of patients receiving neoadjuvant or adjuvant chemotherapy; in more than 95% of cases this was an anthracycline or anthracycline plus taxane combination regimen.

Other limitations of this study included restricting the main cohort to patients aged 40 years or younger at the time of diagnosis to enrich for *BRCA* mutation carriers. It is possible that observations in young-onset breast cancer patients might not translate to older ages at diagnosis. Progesterone-receptor testing was not done routinely in many UK centres during the period of recruitment and supplementary data were derived from tissue microarrays rather than full tumour sections. The relevance of triple-negative breast cancer in terms of biology and treatment has only become apparent since the POSH study was designed, so the study was not powered for this as the primary outcome; notably, the only difference in overall survival in this study was seen between mutation carriers and non-carriers in this subgroup. Recommendations for adjuvant treatment in the UK changed over the course of recruitment, with taxanes being recommended for node-positive disease from 2006 and adjuvant trastuzumab for HER2-positive breast cancer routinely available only from 2006. Although we specifically collected information at 5 years about risk-reducing surgery, we cannot exclude the possibility that risk-reducing mastectomy and oophorectomy might have been done at different hospitals from the recruiting cancer centre (eg, at specialist plastic surgery or gynaecological units).

This study confirmed that patients diagnosed with invasive breast cancer aged 18–40 years have a high breast-cancer-specific mortality, and a high proportion are *BRCA1* and *BRCA2* mutation carriers. We found no clear evidence that either *BRCA1* or *BRCA2* germline mutations significantly affect overall survival with breast cancer after adjusting for known prognostic factors. Decisions about timing of risk-reducing surgery should take into account primary tumour prognosis and patient preference. *BRCA* mutation carriers presenting with triple-negative breast cancer might have an improved survival during the first few years after diagnosis compared with non-carriers, although immediate bilateral mastectomy did not account for this advantage. Finally, analysis of early outcome data from trials exploring *BRCA*-deficient tumour treatment in patients with triple-negative breast cancer should be interpreted with caution in view of the possible early survival advantage for *BRCA* mutation carriers.

For more about the **POSH study** see http://www.southampton.ac.uk/medicine/research/posh.page

For the **BOADICEA algorithm** see http://ccge.medschl.cam.ac.uk/boadicea/
