# Supplementary material for: Germline BRCA mutation and outcome in young-onset breast cancer (POSH): a prospective cohort study
Source: Lancet Oncol. 2018 Feb;19(2):169–80. doi: 10.1016/S1470-2045(17)30891-4 (PMC5805863; doi:10.1016/S1470-2045(17)30891-4)

THE LANCET
Oncology

Supplementary appendix

This appendix formed part of the original submission and has been peer reviewed. We post it as supplied by the authors.

Supplement to: Copson ER, Maishman TC, Tapper WJ, et al. Germline *BRCA* mutation and outcome in young-onset breast cancer (POSH): a prospective cohort study. *Lancet Oncol* 2018; published online Jan 11. http://dx.doi.org/10.1016/S1470-2045(17)30891-4.

Appendix - Tables

Appendix Table 1: Recruitment by active sites

List of recruitment number by all active sites in the reported cohort.

Recruiting Hospital	Principal Investigator	No. recruited
Guys Hospital	Mr. Hisham Hamed	126
Mount Vernon Hospital	Dr. Andreas Makris	108
Royal South Hants Hospital	Dr. Peter Simmonds	91
Weston Park Hospital	Lucy Birch	90
Maidstone Hospital	Dr. Rema Jyothirmayi	89
Royal Stoke University Hospital	Dr. Adrian Murray Brunt	87
Royal Cornwall Hospital	Dr. Duncan Wheatley	80
Royal Free Hospital	Dr. Jackie Newby	77
Queen Alexandra Hospital	Mr. Constantinos Yiangou	73
Ninewells Hospital	Professor A.M.Thompson	68
Southend Hospital	Dr Hafiz Algurafi	63
The Royal Surrey County Hospital	Avril Adams	59
Christie Hospital	Prof. Gareth Evans (Genetics) Dr. Andrew Wardley (Oncology)	53
Wexham Park (formerly Heatherwood & Wexham) Hospital	Dr. Marcia Hall	53
Royal Derby Hospital	Mr. Mark Sibbering	50
The James Cook University Hospital	Dr. John Hardman	50
Frenchay Hospital	Mr. Simon Cawthorn/Dr. Mike Shere	49
Velindre Hospital	Professor Peter Barrett-Lee	45
Belfast City Hospital	Dr. Seamus McAleer	44
Broomfield Hospital	Dr. Saad Tahir	43
Addenbrookes Hospital	Professor Helena Earl	41
The Great Western Hospital	Mr. Marcus Galea	40
Torbay Hospital	Dr. Peter Bliss	38
Countess of Chester Hospital NHS Trust	Mrs Claudia Harding-Mckean	37
Norfolk & Norwich University Hospital NHS Trust	Dr. Adrian Harnett	36
Milton Keynes Hospital NHS Trust	Miss Amanda Taylor	34
Withington Hospital	Dr. Anne Armstrong	32
Royal Marsden Hospital	Prof. Ros Eeles	31
Peterborough Hospital NHS Trust	Dr. Karen McAdam	30
Salisbury Healthcare NHS Trust	Dr. Clare Crowley	30
Manor Hospital	Dr. Inderajit Fernando	29
Royal Berkshire Hospital	Dr Madhumita Bhattachayya	29
The Hillingdon Hospital NHS Trust	Dr. Amy Guppy	29
Hope Hospital	Miss Zahida Saad	27
Macclesfield District General Hospital	Mr. Jalal Kokan	27
Nottingham City Hospital	Mr. R. Douglas Macmillan	27
Glan Clwyd Hospital	Dr. Jill Bishop	26
George Eliot Hospital NHS Trust	Dr. Susan Lupton	25
North Hampshire Hospital	Miss Anne Stebbing	25
Royal Devon and Exeter Hospital	Dr. Anne Hong	25
Royal Bournemouth Hospital	Mr. Anthony Skene	24
Stepping Hill Hospital	Mr. Mohammad Sharif	24
Wrexham Maelor Hospital	Dr Win Soe	24
Isle of Wight NHS Primary Care Trust	Dr. Jenny Marshall	23
Lister Hospital	Dr. Nihal Shah	22
Royal Victoria Infirmary	Dr. Radha Todd	22
Croydon University Hospital (Mayday Hospital)	Dr. Navita Somaiah	21
Royal Sussex County Hospital	Dr. David Bloomfield	21
Surrey & Sussex Heathcare NHS Trust	Miss Shamaela Waheed	21
Whittington Hospital	Prof. Jayant Vaidya	21
Yeovil District Hospital	Dr. G.E Sparrow	21
Barts & The London NHS Trust	Professor Peter Schmid	19
Derriford Hospital	Dr. Steve Kelly	19
Grantham & District Hospital	Mr. Jibril A. Jibril	19
Royal Hampshire County Hospital	Mr. D. Rainsbury	19
Walsgrave Hospital	Professor Robert J Grieve	19
Worthing Hospital	Mr. R. Bonomi	19
Queen's Hospital, Burton	Mr. Colin Rogers	18
St Georges' Hospital	Dr. Laura Assersohn	18
Huddersfield Royal Infirmary	Dr. Jonathan K Joffe	17
Kent & Canterbury Hospital	Dr. Natasha Mithal	17
Poole Hospital NHS Trust	Miss Abigail Evans	17
Stirling Royal Infirmary	Judith Fraser	17
Sunderland Royal Hospital	Mr Obiukwu Iwuchukwu (until 2015)	17
Dorset County Hospital	Sarah Williams	16
North Middlesex University Hospital	Dr. Fharat Raja	16
Royal Albert Edward Infirmary	Dr Elena Takeuchi	16
Solihull Hospital	Dr Medy Tsalic	16
Whipps Cross University Hospital	Mr. Peter Frecker	16

Recruiting Hospital	Principal Investigator	No. recruited
Frimley Park Hospital	Mr. Ian Laidlaw	15
New Cross Hospital	Dr. Rakesh Mehra	15
Royal Liverpool University Hospital	Mr. Chris Holcombe	15
University Hospital of Hartlepool	Mr. Pud Bhaskar	15
Withybush General Hospital	Dr. Gianfilippo Bertelli	15
Darlington Memorial Hospital	Dr. Alison Humphreys	14
Royal Preston Hospital	Dr. Elaine Young	14
Warwick Hospital	Dr. Nawaz Walji	14
William Harvey Hospital	Dr. Natasha Mithal	14
King George Hospital	Dr. Eliot Sims	13
Newham University Hospital NHS Trust	Professor Peter Schmid	13
Russells Hall Hospital	Dr. Rozenn Allerton	13
Charing Cross Hospital	Professor Charles Coombes	12
Darent Valley Hospital	Dr. Julia Hall	12
Friarage Hospital	Dr. Johannes Van Der Voet	12
North Devon District Hospital	Dr. Mark Napier	12
Cumberland Infirmary	Mr. M. Williams	11
The Shrewsbury & Telford Hospital (formerly Royal Shrewsbur)	Dr. Rajiv Agrawal	11
Stoke Mandeville Hospital	Dr. Ketan Shah	11
Wycombe Hospital	Dr. Ketan Shah	11
Kidderminster Hospital	Dr. Mark Churn	10
Queens Hospital (Oldchurch Hospital)	Dr. Mary Quigley	10
Sandwell Hospital	Dr. David Spooner	10
St. Richard's Hospital	Dr. Joanna Gale	10
Stafford General Hospital	Dr. Adrian Murray Brunt	10
Luton & Dunstable Hospital NHS Foundation Trust	Dr. Mei-Lin Ah-See	9
University College London	Dr. Grant Stewart (to 2012)	9
Homerton University Hospital NHS Foundation Trust (c/o Barts)	Professor Peter Schmid	8
James Paget Healthcare NHS Trust	Dr. Adrian Harnett	7
North Tyneside General Hospital	Mr. Mike Carr	7
Queen Elizabeth Hospital, Gateshead	Mr. David Browell	7
Royal Glamorgan Hospital	Dr. Jacinta Abraham	7
Royal Lancaster Infirmary	Dr. David Eaton	7
Royal Oldham	Dr. Juliette Loncaster	7
Birmingham City Hospital	Dr. David Spooner	6
Gwynedd Hospital (North West Wales)	Dr. Jill Bishop	6
Lincoln County Hospital	Mr. Jibril A. Jibril	6
South Tyneside District Hospital	Dr. Radha Todd	6
The Alexandra Hospital	Dr. Clive Irwin	6
The Leeds Teaching Hospital NHS Trust	Dr. Julian Adlard	6
Princess Royal University Hospital	Dr. Mark Harries	5
Wansbeck General Hospital	Mr. Mike Carr	5
West Suffolk Hospital	Dr. Margaret Moody	5
West Wales General	Dr. Margaret Wilkins	5
Conquest Hospital	Dr. Gillian Sadler	4
Royal Alexandra Hospital	Dr. Abdulla Al-hasso	4
Singleton Hospital	Dr. Gianfilippo Bertelli	4
Furness General Hospital	Dr. Geraldine Skailes	3
Queen Elizabeth The Queen Mother Hospital	Dr. Natasha Mithal	3
Bronglais Hospital	Sarah J Jones	2
Burnley General Hospital	Dr. Martin Hogg	2
Kings College London	Dr. Anne Rigg	2
University Hospital of North Tees	Mr. Colm Hennessy	2
Blackburn Royal Infirmary	Dr. Martin Hogg	1
Princess Elizabeth Hospital	Dr. Peter Gomes	1
Queen Elizabeth Hospital, Woolwich	Dr. Hartmut Kristeleit	1
Southern General Hospital	Dr. Abdulla Al-hasso	1

Appendix Table 2: List of BRCA1 and BRCA2 mutation annotation
List of 338 pathogenic BRCA1 and BRCA2 variants included in the BRCA+ group

GENE	Coding change	Protein change
BRCA1	c.514delC	p.Gln172fs
BRCA1	c.1961dupA	p.Lys654fs
BRCA1	c.3762_3763het_delGA	p.Cys1252fs
BRCA1	c.135-1G>T	
BRCA1	c.3400G>T	p.Glu1134X
BRCA1	c.3607C>T	p.Arg1203X
BRCA1	c.53T>C	p.Met18Thr
BRCA1	c.5153G>A	p.Trp1718X
BRCA1	c.302-1G>T	
BRCA1	c.4185+1G>T	
BRCA1	c.2680_2681del	p.Lys894fs
BRCA1	c.69_79del	p.Cys24fs
BRCA1	c.4065_4068delTCAA	p.Asn1355fs
BRCA1	c.4185+1G>T	
BRCA1	c.4357+2T>G	
BRCA1	c.3967C>T	p.Gln1323X
BRCA1	c.4065_4068delTCAA	p.Asn1355fs
BRCA1	c.4180delA	p.Thr1394fs
BRCA1	c.3668_3669insTCCC	p.Leu1223fs
BRCA1	c.1675delA	p.Lys519Argfs
BRCA1	c.427G>T	p.Glu143X
BRCA1	c.4065_4068delTCAA	p.Asn1355fs
BRCA1	c.5503C>T	p.Arg1835X
BRCA1	c.427G>T	p.Glu143X
BRCA1	c.4357+6T>C	
BRCA1	c.1793T>G	p.Leu598X
BRCA1	c.5152+1G>T	
BRCA1	c.1954dupA	p.Lys652fs
BRCA1	c.5152+1G>T	
BRCA1	c.3751_3754delGTCT	p.Cys1252fs
BRCA1	c.3768_3769del	p.Glu1257Glyfs
BRCA1	c.5152+1G>T,	
BRCA1	c.3751_3754delGTCT	p.Cys1252fs
BRCA1	c.A4558T	p.R1520X
BRCA1	c.5194-12G>A	
BRCA1	c.4574_4575delAA	p.Gln1525Argfs
BRCA1	c.5194-12G>A	
BRCA1	c.5332+1G>A	
BRCA1	c.929delA	p.Gln310fs
BRCA1	c.427G>T	p.Glu143X
BRCA1	c.4574_4575delAA	p.Gln1525Argfs
BRCA1	c.5264dupC	p.Ser1755fs
BRCA1	c.1512dupT	p.Arg504fs
BRCA1	c.427G>T	p.Glu143X
BRCA1	c.1266T>G	p.Tyr422X
BRCA1	c.1A>G	p.Met1Val
BRCA1	c.5153G>A	p.Trp1718X
BRCA1	c.1823_1826delAGAA	pLys608fs
BRCA1	c.4586dupT	p.I1529fs
BRCA1	c.4327C>T	p.Arg1443X
BRCA1	c.3751_3754delGTCT	p.Cys1252fs
BRCA1	c.547+2T>A	
BRCA1	c.2068delA	p.Lys690fs
BRCA1	c.2475delC	p.Asp825fs
BRCA1	c.4065_4068delTCAA	p.Asn1355fs
BRCA1	c.4065_4068delTCAA	p.Asn1355fs
BRCA1	c.3331_3334del	p.(Gln1111Asnfs*5)
BRCA1	c.2612_2613insT	p.Pro871fs
BRCA1	c.2074delC	p.His692fs
BRCA1	c.5264dupC	p.Ser1755fs
BRCA1	c.2676_2679del	p.Lys893fs
BRCA1	c.3718C>T	p.Gln1240X
BRCA1	c.5264dupC	p.Ser1755fs
BRCA1	c.1297_1298insCC	p.Ala433fs
BRCA1	c.68-69delAG	p..Glu23Valfs
BRCA1	c.4065_4068delTCAA	p.Asn1355fs
BRCA1	c.181T>G	p.Cys61Gly
BRCA1	c.3751_3754delGTCT	p.Cys1252fs
BRCA1	c.5193delG	p.E1731fs
BRCA1	Deletion exon 1-23	
BRCA1	Deletion exon 1-23	
BRCA1	c.4065_4068delTCAA	p.Asn1355fs

GENE	Coding change	Protein change
BRCA1	c.66dupA	p.Leu22fs
BRCA1	c.68-69delAG	p..Glu23Valfs
BRCA1	c.1141A>T	p.Lys381X
BRCA1	c.2125_2126insA	p.Phe709fs
BRCA1	c.68-69delAG	p..Glu23Valfs
BRCA1	c.5186delT	p.Leu1729fs
BRCA1	c.3228_3229del	p.(Gly1077Alafs*8)
BRCA1	c.68-69delAG	p..Glu23Valfs
BRCA1	c.2676_2679del	p.Lys893fs
BRCA1	Deletion exon 20	
BRCA1	c.4411delG	p.Gly1471fs
BRCA1	c.3331_3334del	p.(Gln1111Asnfs*5)
BRCA1	c.2704delG	p.Glu902fs
BRCA1	Deletion exon 21-24	
BRCA1	c.68-69delAG	p..Glu23Valfs
BRCA1	c.3331_3334delCAAG	p.Gln1111Asnfs
BRCA1	Deletion exon 21-24	
BRCA1	c.3002delA	p.Glu1001fs
BRCA1	c.5054C>T	p.Thr1685Ile
BRCA1	c.4065_4068delTCAA	p.Asn1355fs
BRCA1	c.1012A>T	p.Lys338X
BRCA1	c.3064dupA	p.Thr1022fs
BRCA1	c.5363G>T	p.Gly1788Val
BRCA1	c.303T>G	p.Tyr101X
BRCA1	Deletion of exon 20	
BRCA1	c.69_79del	p.Cys24fs
BRCA1	c.5264dupC	p.Ser1755fs
BRCA1	Deletion of exon 24	
BRCA1	c.520delC	p.Gln174fs
BRCA1	c.2680_2681del	p.Lys894fs
BRCA1	c.427G>T	p.Glu143X
BRCA1	Deletion of exon 3	
BRCA1	c.2680_2681del	p.Lys894fs
BRCA1	Deletion of exon 3	
BRCA1	c.3228_3229del	p.(Gly1077Alafs*8)
BRCA1	c.3400G>T	p.Glu1134X
BRCA1	c.4065_4068delTCAA	p.Asn1355fs
BRCA1	c.4357delG	p.A1453fs
BRCA1	Deletion of exons 1-17	
BRCA1	Deletion of exons 1-17	
BRCA1	Deletion of exons 1-17	
BRCA1	Deletion of exons 1-17	
BRCA1	c.181T>G	p.Cys61Gly
BRCA1	Deletion of exons 1-2	
BRCA1	c.1954dupA	p.Lys652fs
BRCA1	c.1961delA	p.Lys654fs
BRCA1	c.1326T>A	p.Cys442X
BRCA1	c.4354A>T	p.Lys1452X
BRCA1	Deletion of exons 1-2	
BRCA1	Deletion of exons 1-2	
BRCA1	c303T>G	p.Tyr101Ter
BRCA1	c.1954delA	p.Lys652fs
BRCA1	c.2475delC	p.Asp825fs
BRCA1	c.1471C>T	p.Gln491X
BRCA1	c.3751_3754delGTCT	p.Cys1252fs
BRCA1	c.3869_3870delAA	p.Arg1290fs
BRCA1	c.3751_3754delGTCT	p.Cys1252fs
BRCA1	c.68-69delAG	p..Glu23Valfs
BRCA1	c.5251C>T	p.Arg1751Ter
BRCA1	c.5153G>A	p.Trp1718X
BRCA1	c.5503C>T	p.Arg1835X
BRCA1	c.427G>T	p.Glu143X
BRCA1	c.4964_4982del	p.(Ser1655Tyrfs*16)
BRCA1	c.4574_4575delAA	p.Gln1525Argfs
BRCA1	Deletion of exons 14-17	
BRCA1	c.1961dupA	p.Lys654fs
BRCA1	c.1601_1602delAG	p.Gln534fs-X3
BRCA1	Deletion of exons 1a-1b	
BRCA1	c.4065_4068delTCAA	p.Asn1355fs
BRCA1	c.427G>T	p.Glu143X
BRCA1	c.1749_1755del	p.(Lys583Asnfs*3)
BRCA1	Deletion of exons 1a-2	
BRCA1	c.1504_1508del	p.(Leu502Alafs*2)
BRCA1	c.2199delG	p.Glu733fs
BRCA1	Deletion of exons 1A-2	

GENE	Coding change	Protein change
BRCA1	c.5503C>T	p.Arg1835X
BRCA1	Deletion of exons 20	
BRCA1	c.5324T>G	p.Met1775Arg
BRCA1	Deletion of exons 21-24	
BRCA1	c.1949_1950delTA	p.Ile650fs]
BRCA1	c.5264dupC	p.Ser1755fs
BRCA1	c.2267delG	p.Arg756fs
BRCA1	c.5573delT	p.I1858fs
BRCA1	c.5324T>G	p.Met1775Arg
BRCA1	c.4574_4575delAA	p.Gln1525Argfs
BRCA1	Deletion of exons 8-13	
BRCA1	c.4349C>G	p.Ser1450X
BRCA1	c.4106delC	p.Ala1369fs
BRCA1	c.3046_3047insATGAG	p.Asn1016fs
BRCA1	c.3400G>T	p.Glu1134X
BRCA1	c.2953delC	p.Pro985fs
BRCA1	c.187_188delAG	p.Glu23Valfs
BRCA1	Duplication of exon 13	
BRCA1	c.4065_4068delTCAA	p.Asn1355fs
BRCA1	c.68-69delAG	p..Glu23Valfs
BRCA1	c.4165_4166delAG	p.Ser1389X
BRCA1	c.3450_3453delCAAG	p.Gln1111fs
BRCA1	c.981_982del	p.Cys328Terfs
BRCA1	c.427G>T	p.Glu143X
BRCA1	Duplication of exon 13	
BRCA1	c.2068delA	p.Lys690fs
BRCA1	Duplication of exon 13	
BRCA1	c.3400G>T	p.Glu1134X
BRCA1	c.3751_3754delGTCT	p.Cys1252fs
BRCA1	c.5503C>T	p.Arg1835X
BRCA1	c.797_798del	p.Val266fs
BRCA1	c.675delT	p.Ala225fs
BRCA1	Duplication of exon 13	
BRCA1	Duplication of exon 13	
BRCA1	c.929delA	p.Gln310fs
BRCA1	c.4065_4068delTCAA	p.Asn1355fs
BRCA1	c.1756delC	p.Pro586fs
BRCA1	c.181T>G	p.Cys61Gly
BRCA1	Duplication of exon 13	
BRCA1	Duplication of exon 13	
BRCA1	c.3331_3334del	p.(Gln1111Asnfs*5)
BRCA1	c.929delA	p.Gln310fs
BRCA1	c.1823_1826delAGAA	pLys608fs
BRCA1	Duplication of exon 13	
BRCA1	c.3751_3754delGTCT	p.Cys1252fs
BRCA1	c.68-69delAG	p..Glu23Valfs
BRCA1	Duplication of exon 13	
BRCA1	c.427G>T	p.Glu143X
BRCA1	c.5027T>A	p.Leu1676X
BRCA1	Duplication of exon 13	
BRCA1	Duplication of exon 5-8	
BRCA1	c.1823_1826delAGAA	p.Lys608fs
BRCA1	c.4065_4068delTCAA	p.Asn135Lysfs
BRCA1	c.5095C>T	p.Arg1699Trp
BRCA2	c.1813delA	p.Ile605fs
BRCA2	c.2330dupA	p.Asp777fs
BRCA2	c.1813delA	p.Ile605fs
BRCA2	c.5909C>A	p.Ser1970X
BRCA2	c.7762delA	p.Ile2588fs
BRCA2	c.4398_4402del	p.Leu1466Phefs
BRCA2	c.7757G>A	p.Trp2586X
BRCA2	c.7480C>T	p.Arg2494X
BRCA2	c.5946delT	p.Ser1982fs
BRCA2	c.9154C>T	p.Arg3052Trp
BRCA2	c.7542G>T	p.Gly2439X
BRCA2	c.8395delA	p.Arg2799fs
BRCA2	c.517-2A>G	
BRCA2	c.5130_5133del	p.Tyr1710fs-X
BRCA2	c.755_758del	p.Asp252Valfs
BRCA2	c.517-2A>G	
BRCA2	c.7988A>T	p.Glu2663Val
BRCA2	c.4416_4419del	p.(Asn1473Lysfs*5)
BRCA2	c.3785C>G	p.Ser1262X
BRCA2	c.4729G>T	p.Glu1577X
BRCA2	c.4972C>T	p.Gln1658X

GENE	Coding change	Protein change
BRCA2	c.5682C>G	p.Tyr1894X
BRCA2	c.274C>T	p.Gln92X
BRCA2	c.7654dupA	p.Ile2552fs
BRCA2	c.6275_6276del	p.Leu2093fs
BRCA2	c.6405_6409del	p.(Asn2135Lysfs*3)
BRCA2	c.8940dupA	p.Glu2981Argfs
BRCA2	c.9382C>T	p.Arg3128X
BRCA2	c.5682C>G	p.Tyr1894X
BRCA2	c.6275_6276del	p.Leu2093fs
BRCA2	c.7884dupA	p.Trp2629fs
BRCA2	c.1813dupA	p.Ile605fs
BRCA2	c.4478_4481delAAAG	p.Glu1493Valfs
BRCA2	c.4478_4481delAAAG	p.Glu1493Valfs
BRCA2	c.3847_3848delGT	p.Val1283fs
BRCA2	c.6757_6758del	p.(Leu2253Phefs*7)
BRCA2	c.9382C>T	p.Arg3128X
BRCA2	c.5303_5304delTT	p.Leu1768Argfs
BRCA2	c.7977-1G>C	
BRCA2	c.8755-1G>A	
BRCA2	c.1705_1706del	p.(Gln569Glufs*20)
BRCA2	c.9357_9360del	p.(Ile3120Leufs*42)
BRCA2	c.439C>T	p.Gln147X
BRCA2	c.9182delT	p.Leu3061X
BRCA2	c.7762delA	p.Ile2588fs
BRCA2	c.6275_6276del	p.Leu2093fs
BRCA2	Deletion exon 21	
BRCA2	c.3969_3970insCAAA	p.Lys1323fs
BRCA2	c.4478_4481delAAAG	p.Glu1493Valfs
BRCA2	c.7737_7749delACAGTTGGCTGAT	p.(Ile2579Metfs*65)
BRCA2	c.6275_6276del	p.Leu2093fs
BRCA2	c.6944_6947del	p.Ile2315Lysfs
BRCA2	Deletion exons 14-16	
BRCA2	c.1376T>G	p.Leu459X
BRCA2	c.6275_6276del	p.Leu2093fs
BRCA2	Deletion of exon 17	
BRCA2	c.3847_3848delGT	p.Val1283fs
BRCA2	c.5577_5580del	p.(Lys1861*)
BRCA2	c.1296_1297del	p.(Asn433Glnfs*18)
BRCA2	c.1888dupA	p.Thr630fs
BRCA2	c.8813dup	p.(Asp2938Glufs*2)
BRCA2	c.5682C>G	p.Tyr1894X
BRCA2	c.3248delA	p.Asn1083fs
BRCA2	c.5722_5723del	p.Leu1908fs
BRCA2	c.4478_4481delAAAG	p.Glu1493Valfs
BRCA2	c.8904delC	p.Thr2968fs
BRCA2	c.7757G>A	p.Trp2586X
BRCA2	Deletion of exon 3a	
BRCA2	Deletion of exons 1-11	0
BRCA2	c.755_758del	p.Asp252Valfs
BRCA2	c.5864C>A	p.Ser1955X
BRCA2	c.8904delC	p.Thr2968fs
BRCA2	c.9196C>T	p.Gln3066X
BRCA2	Deletion of exons 1-2	
BRCA2	c.407delA	p.Asn136fs
BRCA2	c.5350_5351delAA	p.Asn1784Hisfs
BRCA2	Deletion of exons 14 - 16	
BRCA2	c.6275_6276del	p.Leu2093fs
BRCA2	Deletion of exons 14-16	
BRCA2	c.3689delC	p.Ser1230fs
BRCA2	c.9435_9436del	p.Ser3147Cysfs
BRCA2	c.7069_7070del	p.Leu2357Valfs
BRCA2	c.5722_5723delCT	p.Leu1908fs
BRCA2	Deletion of exons 14-16	
BRCA2	c.8878C>T	p.Gln2960X
BRCA2	c.8297delC	p.Thr2766fs
BRCA2	c.1813delA	p.Ile605fs
BRCA2	c.5682C>G	p.Tyr1894X
BRCA2	c.6099delA	p.Ile2033fs
BRCA2	c.6079dupA	p.Arg2027fs
BRCA2	c.8297delC	p.Thr2766fs
BRCA2	c.539_540insAT	p.Ile180fs
BRCA2	c.2034_2038delTAATA	p.Asn678fs
BRCA2	c.9382C>T	p.Arg3128X
BRCA2	c.2836_2837del	p.(Asp946Phefs*12)
BRCA2	c.7069_7070del	p.Leu2357Valfs

GENE	Coding change	Protein change
BRCA2	c.8904delC	p.Thr2968fs
BRCA2	c.370dupA	p.Met124fs
BRCA2	c.7007G>A	p.Arg2336His
BRCA2	c.2808_2811del	p.(Ala938Profs*21)
BRCA2	c.5350_5353del	p.Asn1784Hisfs
BRCA2	c.6275_6276del	p.Leu2093fs
BRCA2	c.5682C>G	p.Tyr1894X
BRCA2	c.5946delT	p.Ser1982fs
BRCA2	c.9945delA	p.Lys3315fs
BRCA2	c.6275_6276del	p.Leu2093fs
BRCA2	Deletion of exons 8-10	
BRCA2	c.7480C>T	p.Arg2494X
BRCA2	c.8167G>C	p.Asp2723His
BRCA2	c.7934delG	p.Arg2645fs
BRCA2	c.6816_6820del	p.Gly2274fs
BRCA2	c.1189_1190insTTAG	p.Gln397fs
BRCA2	c.755_758del	p.Asp252Valfs
BRCA2	c.9117G>A	p.Pro3039Pro
BRCA2	c.5946delT	p.Ser1982fs
BRCA2	c.755_758del	p.Asp252Valfs
BRCA2	c.9972A>T	p.Lys3326X
BRCA2	c.3405C>A	p.Tyr1135X
BRCA2	c.4478_4481delAAAG	p.Glu1493Valfs
BRCA2	c.574_575del	p.(Met192Valfs*13)
BRCA2	c.6275_6276del	p.Leu2093fs
BRCA2	c.5645C>A	p.Ser1882X
BRCA2	c.3785C>G	p.Ser1262X
BRCA2	c.9196C>T	p.Gln3066X
BRCA2	c.6643delT	p.Tyr2215fs
BRCA2	c.755_758del	p.Asp252Valfs
BRCA2	c.6275_6276del	p.Leu2093fs
BRCA2	c.4169delT	p.Leu1390fs
BRCA2	c.9382C>T	p.Arg3128X
BRCA2	c.5350_5351delAA	p.Asn1784Hisfs
BRCA2	c.396T>A	p.Cys132X
BRCA2	c.1389_1390del	p.463_464del
BRCA2	c.5350_5351delAA	p.Asn1784Hisfs
BRCA2	c.5682C>G	p.Tyr1894X
BRCA2	c.6333_6337del	p.(Arg2112Profs*15)
BRCA2	c.1459delA	p.Ile411Tyrfs

Appendix Table 3: Cause of death breakdown by BRCA status (analysis population who died)
List of all causes of death in the reported cohort.

**Characteristic**	**All patients (n=678)**	**BRCA1+ (n=47)**	**BRCA2+ (n=37)**	**BRCA+ (n=84)**	**BRCA- (n=594)**
Cause of death					
**Breast Cancer**	**651 (96·0%)**	**41 (87·2%)**	**36 (97·3%)**	**77 (91·7%)**	**574 (96·6%)**
**Other Cancer**	**18 (2·7%)**	**6 (12·8%)**	**0 (0%)**	**6 (7·1%)**	**12 (2%)**
Brain	1 (0·1%)	0 (0%)	0 (0%)	0 (0%)	1 (0·2%)
Colorectal	1 (0·1%)	0 (0%)	0 (0%)	0 (0%)	1 (0·2%)
Gastric	1 (0·1%)	0 (0%)	0 (0%)	0 (0%)	1 (0·2%)
Haematological	4 (0·6%)	0 (0%)	0 (0%)	0 (0%)	4 (0·7%)
Lung	3 (0·4%)	0 (0%)	0 (0%)	0 (0%)	3 (0·5%)
Oesophageal	1 (0·1%)	1 (2·1%)	0 (0%)	1 (1·2%)	0 (0%)
Ovarian	3 (0·4%)	3 (6·4%)	0 (0%)	3 (3·6%)	0 (0%)
Pancreas	1 (0·1%)	1 (2·1%)	0 (0%)	1 (1·2%)	0 (0%)
Pancreatic	1 (0·1%)	0 (0%)	0 (0%)	0 (0%)	1 (0·2%)
Peritoneal	1 (0·1%)	1 (2·1%)	0 (0%)	1 (1·2%)	0 (0%)
Sarcoma	1 (0·1%)	0 (0%)	0 (0%)	0 (0%)	1 (0·2%)
**Other**	**8 (1·2%)**	**0 (0%)**	**1 (2·7%)**	**1 (1·2%)**	**7 (1·2%)**
Accident	1 (0·1%)	0 (0%)	0 (0%)	0 (0%)	1 (0·2%)
Adrenal insufficiency	1 (0·1%)	0 (0%)	0 (0%)	0 (0%)	1 (0·2%)
Alcohol	2 (0·3%)	0 (0%)	0 (0%)	0 (0%)	2 (0·3%)
Alcohol, adrenal failure	1 (0·1%)	0 (0%)	0 (0%)	0 (0%)	1 (0·2%)
Cardiac	1 (0·1%)	0 (0%)	0 (0%)	0 (0%)	1 (0·2%)
Cerebal complication from Crohn's disease	1 (0·1%)	0 (0%)	0 (0%)	0 (0%)	1 (0·2%)
Infection	1 (0·1%)	0 (0%)	1 (2·7%)	1 (1·2%)	0 (0%)
**Unknown**	**1 (0·1%)**	**0 (0%)**	**0 (0%)**	**0 (0%)**	**1 (0·2%)**
Died abroad	1 (0·1%)	0 (0%)	0 (0%)	0 (0%)	1 (0·2%)

Appendix Table 4: Multivariable Analyses - Complete-Case Results (analysis population)

Breakdown of compete-case results for each multivariable analysis carried out on the analysis population.

Characteristic	OS by BRCA		DDFS by BRCA		OS by BRCA1		OS by BRCA2		OS by BRCA (adjusted for time to blood draw)	
	# (events)	HR (95% CI), p-value}	# (events)	HR (95% CI), p-value}	# (events)	HR (95% CI), p-value}	# (events)	HR (95% CI), p-value}	# (events)	HR (95% CI), p-value}
BRCA- (Ref.)	2395 (594)	1.00 (Ref.)	2395 (659)	1.00 (Ref.)	2395 (594)	1.00 (Ref.)	2395 (594)	1.00 (Ref.)	2395 (594)	1.00 (Ref.)
UVA BRCA*+	338 (84)	0.99 (0.78, 1.24), 0.90	338 (93)	0.99 (0.80, 1.23), 0.94	201 (47)	0.93 (0.69, 1.25), 0.64	137 (37)	1.07 (0.76, 1.49), 0.71	338 (84)	1.01 (0.81, 1.27), 0.91
MVA BRCA*+	338 (84)	0.87 (0.66, 1.13), 0.29	338 (93)	0.91 (0.70, 1.17), 0.45	201 (47)	0.86 (0.61, 1.20), 0.37	137 (37)	0.86 (0.58, 1.29), 0.47	338 (84)	0.89 (0.68, 1.17), 0.41
Age at diagnosis	2733 (678)	0.97 (0.95, 1.00), 0.019	2733 (752)	0.97 (0.95, 0.99), 0.014	2596 (641)	0.97 (0.95, 1.00), 0.027	2532 (631)	0.97 (0.95, 1.00), 0.024	2733 (678)	0.97 (0.95, 1.00), 0.018
BMI<25 (Ref.)	1427 (313)	1.00 (Ref.)	1427 (359)	1.00 (Ref.)	1357 (298)	1.00 (Ref.)	1313 (294)	1.00 (Ref.)	1427 (313)	1.00 (Ref.)
25{&le}BMI<30	714 (197)	1.24 (1.02, 1.50), 0.032	714 (211)	1.17 (0.97, 1.41), 0.10	673 (183)	1.20 (0.98, 1.47), 0.077	667 (181)	1.18 (0.97, 1.45), 0.11	714 (197)	1.24 (1.02, 1.51), 0.028
BMI{&ge}30	491 (152)	1.28 (1.03, 1.60), 0.026	491 (166)	1.26 (1.02, 1.55), 0.031	469 (145)	1.26 (1.00, 1.57), 0.046	460 (142)	1.20 (0.96, 1.52), 0.11	491 (152)	1.28 (1.03, 1.60), 0.026
Grade 1 (Ref.)	156 (11)	1.00 (Ref.)	156 (18)	1.00 (Ref.)	156 (11)	1.00 (Ref.)	154 (10)	1.00 (Ref.)	156 (11)	1.00 (Ref.)
Grade 2	904 (200)	2.56 (1.05, 6.25), 0.040	904 (231)	1.67 (0.85, 3.28), 0.13	864 (185)	2.47 (1.01, 6.03), 0.048	888 (197)	2.54 (1.04, 6.21), 0.041	904 (200)	2.58 (1.06, 6.30), 0.038
Grade 3	1598 (450)	3.63 (1.49, 8.83), 0.0045	1598 (482)	2.25 (1.15, 4.39), 0.018	1509 (431)	3.65 (1.50, 8.90), 0.0043	1419 (408)	3.57 (1.47, 8.70), 0.0051	1598 (450)	3.63 (1.49, 8.83), 0.0045
Max. inv. size (cm)	2577 (638)	1.10 (1.06, 1.14), <0.0001	2577 (710)	1.11 (1.07, 1.15), <0.0001	2454 (607)	1.10 (1.06, 1.14), <0.0001	2386 (594)	1.10 (1.06, 1.14), <0.0001	2577 (638)	1.10 (1.06, 1.14), <0.0001
HER2- (Ref.)	1763 (442)	1.00 (Ref.)	1763 (484)	1.00 (Ref.)	1652 (414)	1.00 (Ref.)	1599 (400)	1.00 (Ref.)	1763 (442)	1.00 (Ref.)
HER2+	649 (193)	0.97 (0.80, 1.17), 0.74	649 (218)	1.07 (0.89, 1.28), 0.48	635 (185)	0.94 (0.78, 1.14), 0.56	637 (191)	0.97 (0.80, 1.18), 0.76	649 (193)	0.98 (0.81, 1.18), 0.81
N0 stage (Ref.)	1304 (189)	1.00 (Ref.)	1304 (212)	1.00 (Ref.)	1249 (179)	1.00 (Ref.)	1175 (166)	1.00 (Ref.)	1304 (189)	1.00 (Ref.)
N1 stage	1388 (479)	2.26 (1.84, 2.78), <0.0001	1388 (530)	2.30 (1.90, 2.80), <0.0001	1308 (452)	2.30 (1.86, 2.83), <0.0001	1316 (455)	2.27 (1.83, 2.81), <0.0001	1388 (479)	2.28 (1.86, 2.80), <0.0001
ER- (Ref.)	908 (248)	1.00 (Ref.)	908 (260)	1.00 (Ref.)	887 (245)	1.00 (Ref.)	757 (212)	1.00 (Ref.)	908 (248)	1.00 (Ref.)
ER+ (2 years)	1811 (428)	0.34 (0.25, 0.45), <0.0001	1811 (490)	0.63 (0.52, 0.78), <0.0001	1696 (394)	0.34 (0.25, 0.45), <0.0001	1762 (417)	0.32 (0.23, 0.43), <0.0001	1811 (428)	0.34 (0.25, 0.45), <0.0001
ER+ (5 years)	1811 (428)	1.27 (0.97, 1.67), 0.082	1811 (490)	1.61 (1.23, 2.10), 0.00048	1696 (394)	1.20 (0.93, 1.55), 0.17	1762 (417)	1.21 (0.92, 1.59), 0.17	1811 (428)	1.28 (0.97, 1.69), 0.076
ER+ (10 years)	1811 (428)	2.17 (1.50, 3.13), <0.0001	1811 (490)	3.46 (2.01, 5.95), <0.0001	1696 (394)	2.22 (1.52, 3.27), <0.0001	1762 (417)	2.39 (1.58, 3.61), <0.0001	1811 (428)	2.15 (1.49, 3.10), <0.0001
White ethnicity (Ref.)	2494 (610)	1.00 (Ref.)	2494 (672)	1.00 (Ref.)	2372 (577)	1.00 (Ref.)	2316 (566)	1.00 (Ref.)	2494 (610)	1.00 (Ref.)
Black ethnicity	103 (38)	1.36 (0.94, 1.98), 0.10	103 (44)	1.54 (1.09, 2.18), 0.014	97 (36)	1.41 (0.97, 2.06), 0.075	93 (36)	1.45 (1.00, 2.12), 0.053	103 (38)	1.36 (0.94, 1.97), 0.10
Asian ethnicity	80 (20)	1.01 (0.59, 1.72), 0.98	80 (24)	1.13 (0.70, 1.84), 0.61	76 (20)	1.03 (0.60, 1.76), 0.91	75 (19)	1.00 (0.57, 1.74), 01	80 (20)	0.99 (0.58, 1.69), 0.97
Other ethnicity	21 (3)	0.96 (0.31, 3.01), 0.95	21 (5)	1.18 (0.44, 3.17), 0.74	19 (2)	0.69 (0.17, 2.78), 0.60	18 (3)	1.01 (0.32, 3.17), 0.98	21 (3)	0.99 (0.32, 3.10), 0.99
No use of taxanes (Ref.)	1780 (455)	1.00 (Ref.)	1780 (507)	1.00 (Ref.)	1689 (436)	1.00 (Ref.)	1633 (422)	1.00 (Ref.)	1780 (455)	1.00 (Ref.)
Use of taxanes	659 (190)	1.02 (0.84, 1.23), 0.84	659 (205)	0.95 (0.79, 1.14), 0.56	624 (175)	1.00 (0.83, 1.22), 0.97	614 (177)	1.01 (0.83, 1.23), 0.94	659 (190)	1.01 (0.83, 1.22), 0.95

Appendix Table 5: Multivariable Analyses - Complete-Case Results (TNBC population)
Breakdown of compete-case results for each multivariable analysis carried out on the TNBC population.

Characteristic	OS by BRCA		DDFS by BRCA		OS by BRCA (excluding bilateral mastectomies)		OS by BRCA (excluding new primary or ovarian cancers)	
	# (events)	HR (95% CI), p-value}	# (events)	HR (95% CI), p-value}	# (events)	HR (95% CI), p-value}	# (events)	HR (95% CI), p-value}
BRCA- (Ref.)	422 (120)	1.00 (Ref.)	422 (122)	1.00 (Ref.)	412 (119)	1.00 (Ref.)	407 (114)	1.00 (Ref.)
UVA BRCA+ (at 2 years)	136 (33)	0.59 (0.35, 0.99), 0.044	136 (37)	0.82 (0.55, 1.20), 0.31	115 (27)	0.55 (0.32, 0.97), 0.039	114 (23)	0.60 (0.34, 1.05), 0.071
UVA BRCA+ (at 5 years)	136 (33)	1.09 (0.67, 1.75), 0.75	136 (37)	1.46 (0.81, 2.64), 0.20	115 (27)	1.00 (0.60, 1.68), 0.99	114 (23)	0.80 (0.44, 1.43), 0.46
UVA BRCA+ (at 10 years)	136 (33)	1.96 (0.76, 5.05), 0.17	136 (37)	2.41 (0.83, 7.05), 0.11	115 (27)	1.72 (0.64, 4.63), 0.29	114 (23)	1.08 (0.34, 3.46), 0.90
MVA BRCA+ (at 2 years)	136 (33)	0.51 (0.29, 0.90), 0.019	136 (37)	0.94 (0.50, 1.75), 0.85	115 (27)	0.43 (0.22, 0.80), 0.0084	114 (23)	0.52 (0.28, 0.96), 0.037
MVA BRCA+ (at 5 years)	136 (33)	1.08 (0.65, 1.79), 0.79	136 (37)	1.27 (0.69, 2.35), 0.46	115 (27)	0.90 (0.52, 1.57), 0.73	114 (23)	0.87 (0.47, 1.60), 0.67
MVA BRCA+ (at 10 years)	136 (33)	2.10 (0.80, 5.54), 0.13	136 (37)	3.60 (0.89, 14.49),0 .071	115 (27)	1.72 (0.62, 4.81), 0.30	114 (23)	1.36 (0.44, 4.19), 0.60
Age at diagnosis	558 (153)	1.02 (0.97, 1.08), 0.36	558 (159)	1.02 (0.97, 1.07), 0.48	517 (143)	1.03 (0.98, 1.09), 0.22	521 (137)	1.04 (0.99, 1.10), 0.16
BMI<25 (Ref.)	274 (63)	1.00 (Ref.)	274 (68)	1.00 (Ref.)	257 (60)	1.00 (Ref.)	257 (57)	1.00 (Ref.)
25{&le}BMI<30	149 (54)	1.51 (1.02, 2.23), 0.038	149 (55)	1.41 (0.97, 2.06), 0.074	141 (50)	1.48 (0.99, 2.20), 0.055	139 (50)	1.59 (1.06, 2.37), 0.025
BMI{&ge}30	123 (33)	1.11 (0.71, 1.74), 0.63	123 (33)	0.97 (0.62, 1.50), 0.88	119 (33)	1.10 (0.70, 1.72), 0.68	113 (27)	1.07 (0.66, 1.72), 0.79
Max. inv. size (cm)	523 (143)	1.11 (1.04, 1.19), 0.0012	523 (149)	1.12 (1.05, 1.20), 0.0010	495 (137)	1.11 (1.04, 1.19), 0.0012	491 (130)	1.11 (1.04, 1.19), 0.0014
N0 stage (Ref.)	341 (58)	1.00 (Ref.)	341 (61)	1.00 (Ref.)	322 (55)	1.00 (Ref.)	322 (51)	1.00 (Ref.)
N1 stage	211 (94)	2.72 (1.88, 3.94), <0.0001	211 (97)	2.61 (1.82, 3.75), <0.0001	200 (90)	2.82 (1.93, 4.12), <0.0001	194 (86)	2.98 (2.01, 4.41), <0.0001
White ethnicity (Ref.)	500 (140)	1.00 (Ref.)	500 (145)	1.00 (Ref.)	474 (133)	1.00 (Ref.)	470 (128)	1.00 (Ref.)
Black ethnicity	26 (10)	2.12 (1.02, 4.39), 0.044	26 (11)	2.00 (1.00, 3.97), 0.049	24 (10)	2.52 (1.21, 5.24), 0.014	21 (6)	1.89 (0.82, 4.38), 0.13
Asian ethnicity	19 (1)	0.33 (0.05, 2.36), 0.27	19 (1)	0.28 (0.04, 2.04), 0.21	18 (1)	0.34 (0.05, 2.46), 0.29	18 (1)	0.35 (0.05, 2.49), 0.29
Other ethnicity	5 (1)	0.68 (0.09, 4.90), 0.70	5 (1)	0.96 (0.13, 6.97), 0.97	3 (1)	0.76 (0.10, 5.53), 0.79	5 (1)	0.70 (0.10, 5.08), 0.72
No use of taxanes (Ref.)	384 (98)	1.00 (Ref.)	384 (102)	1.00 (Ref.)	361 (94)	1.00 (Ref.)	357 (88)	1.00 (Ref.)
Use of taxanes	161 (55)	1.17 (0.81, 1.68), 0.41	161 (57)	1.19 (0.84, 1.71), 0.33	154 (52)	1.12 (0.77, 1.64), 0.55	152 (49)	1.12 (0.76, 1.64), 0.57

Appendix - Figures

Appendix Figure 1 – Flow diagram of the POSH cohort
Flow diagram of the POSH cohort.

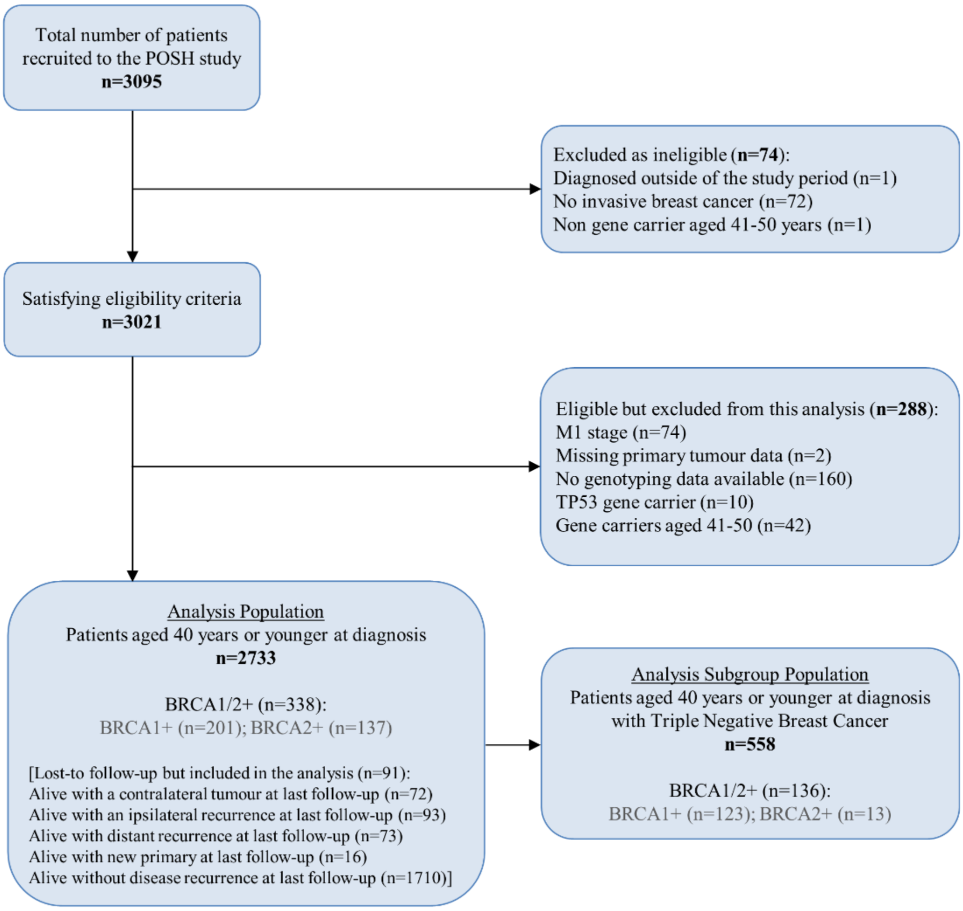

Appendix Figure 2 – Distant Disease Free Survival by *BRCA* status for all patients (analysis population)
Kaplan-Meier plot by *BRCA*1 and/or 2 status (*BRCA*+/-) for Distant Disease Free Survival (OS) (Panel A); and Forest Plot of corresponding univariable and multivariable hazard ratios by *BRCA*+/- status for Distant Disease Free (Panel B). In Panel B, multivariable analysis is adjusted for age, body mass index, grade, tumour size, HER2 status, ER status, ethnicity and use of taxane chemotherapy.

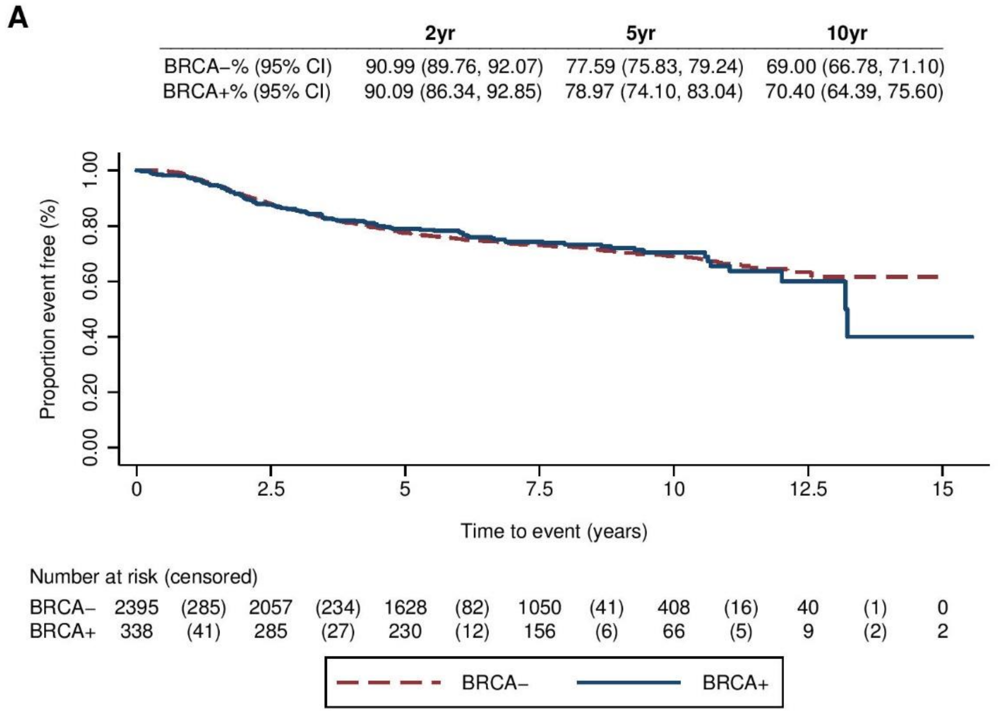

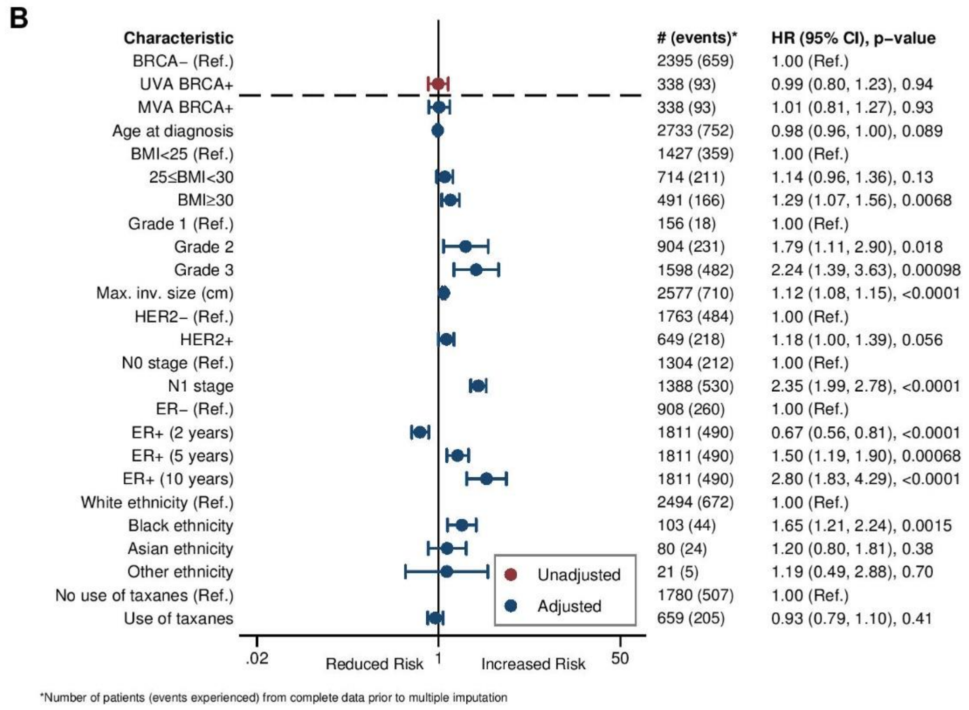

Appendix Figure 3 – Overall Survival by *BRCA1* status for all patients (analysis population)
Kaplan-Meier plot by *BRCA1* status (*BRCA1*+/-) for Overall Survival (OS) (Panel A); and Forest Plot of corresponding univariable and multivariable hazard ratios by *BRCA1*+/- status for Overall Survival (Panel B). In Panel B, multivariable analysis is adjusted for age, body mass index, grade, tumour size, HER2 status, ER status, ethnicity and use of taxane chemotherapy.

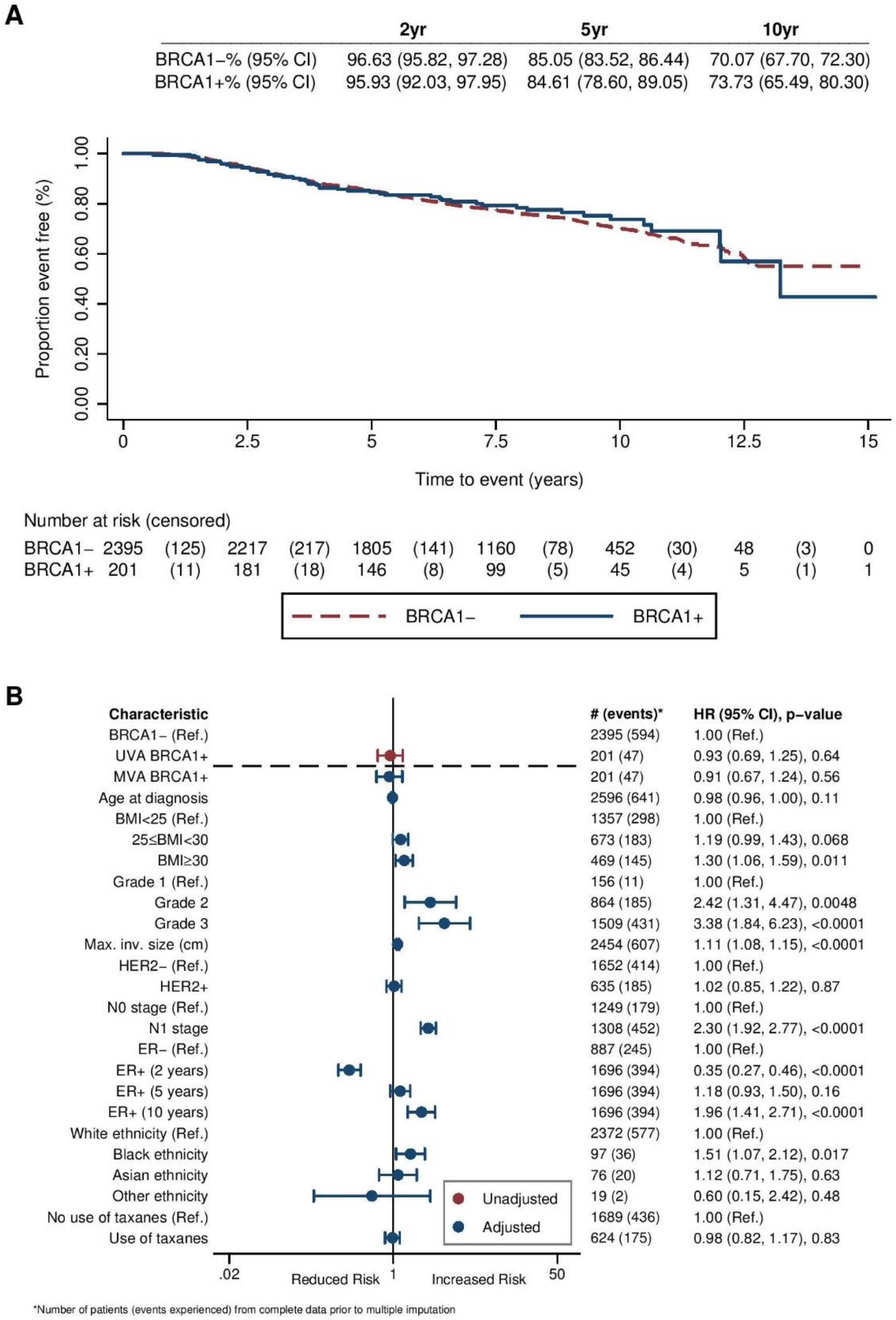

Appendix Figure 4 – Overall Survival by *BRCA2* status for all patients (analysis population)
Kaplan-Meier plot by *BRCA2* status (*BRCA2*+/-) for Overall Survival (OS) (Panel A); and Forest Plot of corresponding univariable and multivariable hazard ratios by *BRCA2*+/- status for Overall Survival (Panel B). In Panel B, multivariable analysis is adjusted for age, body mass index, grade, tumour size, HER2 status, ER status, ethnicity and use of taxane chemotherapy.

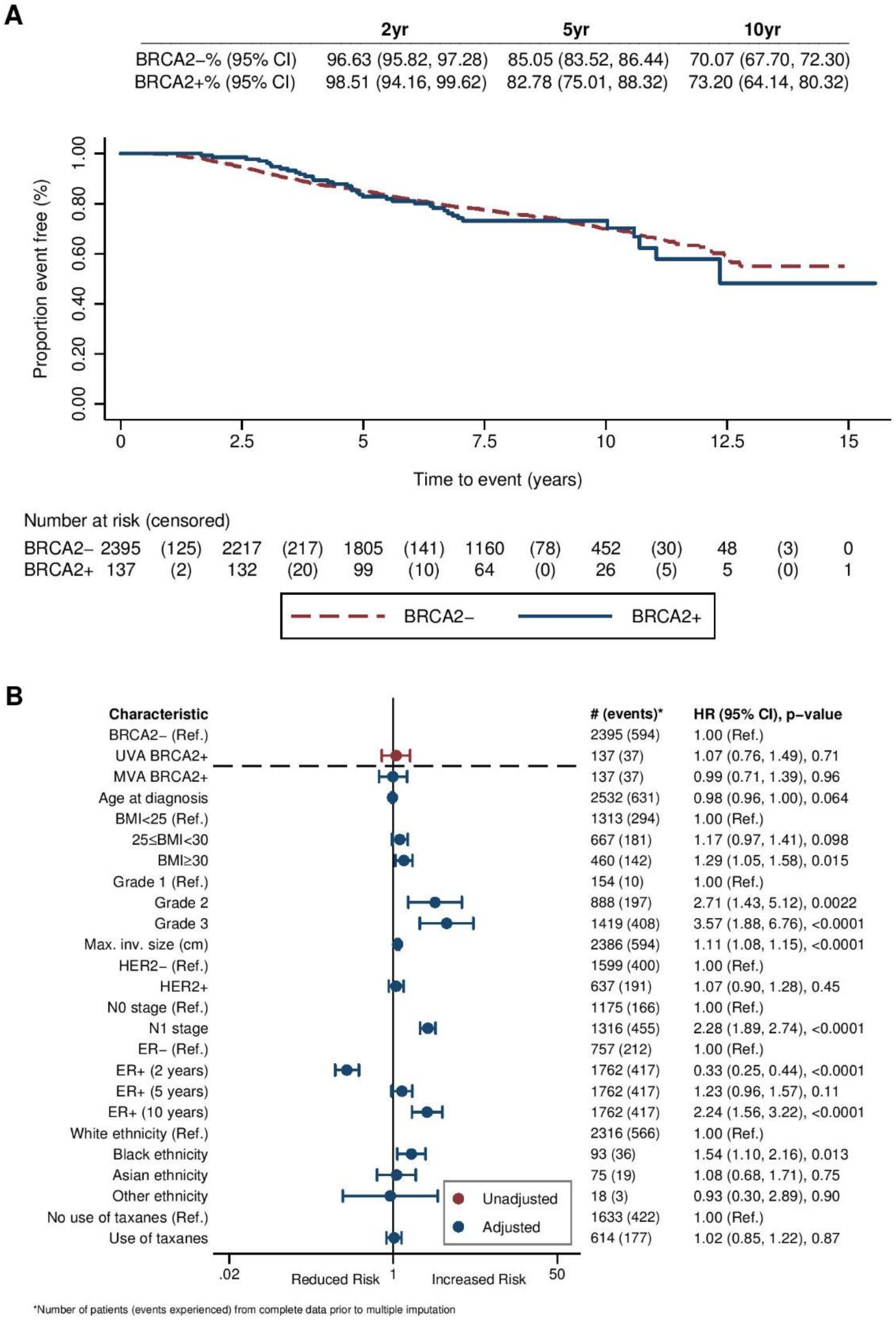

Appendix Figure 5 – Distant Disease Free Survival by *BRCA* status for all TNBC patients (TNBC population)
Kaplan-Meier plot by *BRCA*1 and/or 2 status (*BRCA*+/-) for Distant Disease Free Survival (OS) (Panel A); and Forest Plot of corresponding univariable and multivariable hazard ratios by *BRCA*+/- status for Distant Disease Free Survival (Panel B). In Panel B, multivariable analysis is adjusted for age, body mass index, tumour size, ethnicity and use of taxane chemotherapy.

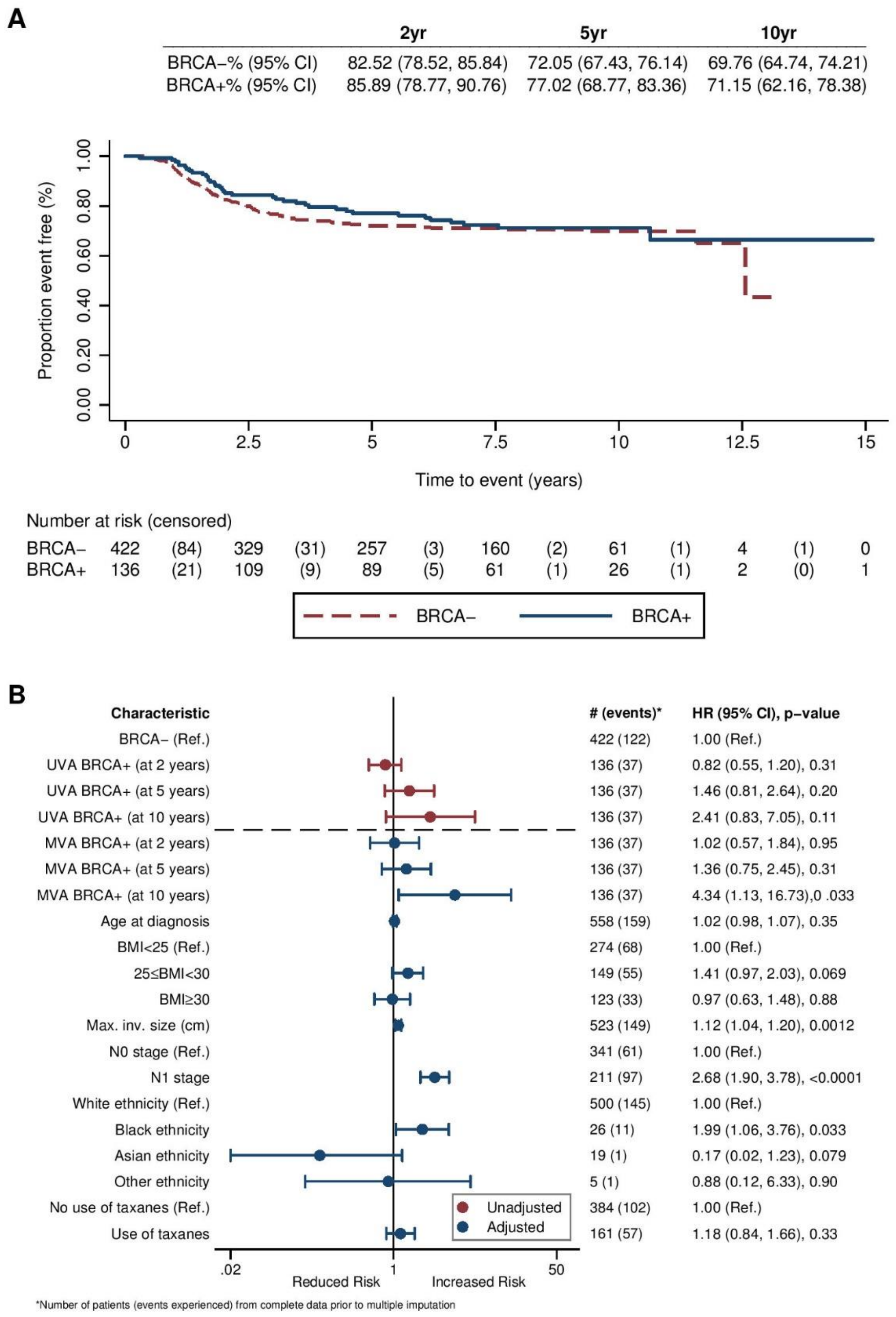

Appendix Figure 6 – Overall Survival by *BRCA* status for all patients, adjusting for time to blood draw (analysis population)

Forest Plot of univariable and multivariable hazard ratios by *BRCA*+/- status for Overall Survival (OS), adjusting for time to blood draw. Multivariable analysis is also adjusted for age, body mass index, grade, tumour size, HER2 status, ER status, ethnicity and use of taxane chemotherapy.

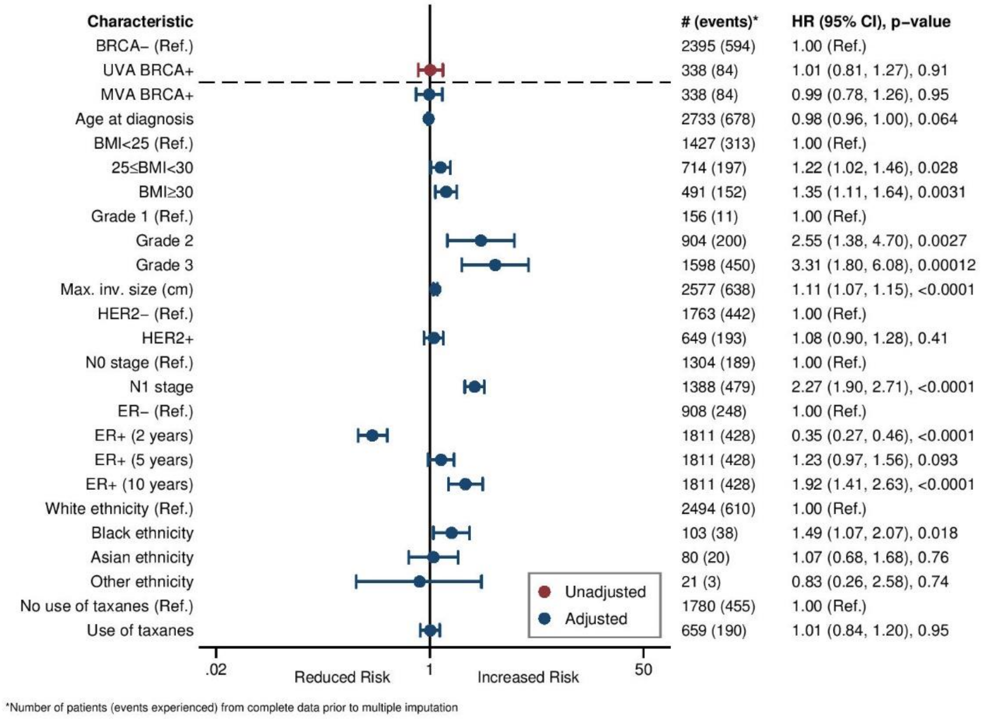

Appendix Figure 7 – Multivariable Analyses - Proportional hazards tests

Proportional hazards (PH) test results for the main comparators for: (A) Overall Survival (OS) by BRCA status – analysis population (PH assumption met); (B) Distant disease free survival (DDFS) by BRCA status – analysis population (PH assumption met); (C) OS by BRCA1 status – analysis population (PH assumption met); (D) OS by BRCA2 status – analysis population (PH assumption met); (E) OS by BRCA status – TNBC population (PH assumption not met); (F) DDFS by BRCA status – TNBC population (PH assumption not met); (G) OS by BRCA status, adjusted for time to blood draw – analysis population (PH assumption met); (H) OS by BRCA status - TNBC population, excluding patients not having immediate bilateral mastectomies (PH assumption not met); (I) OS by BRCA status - TNBC population, excluding patients who developed a new primary breast or ovarian cancer (PH assumption not met).

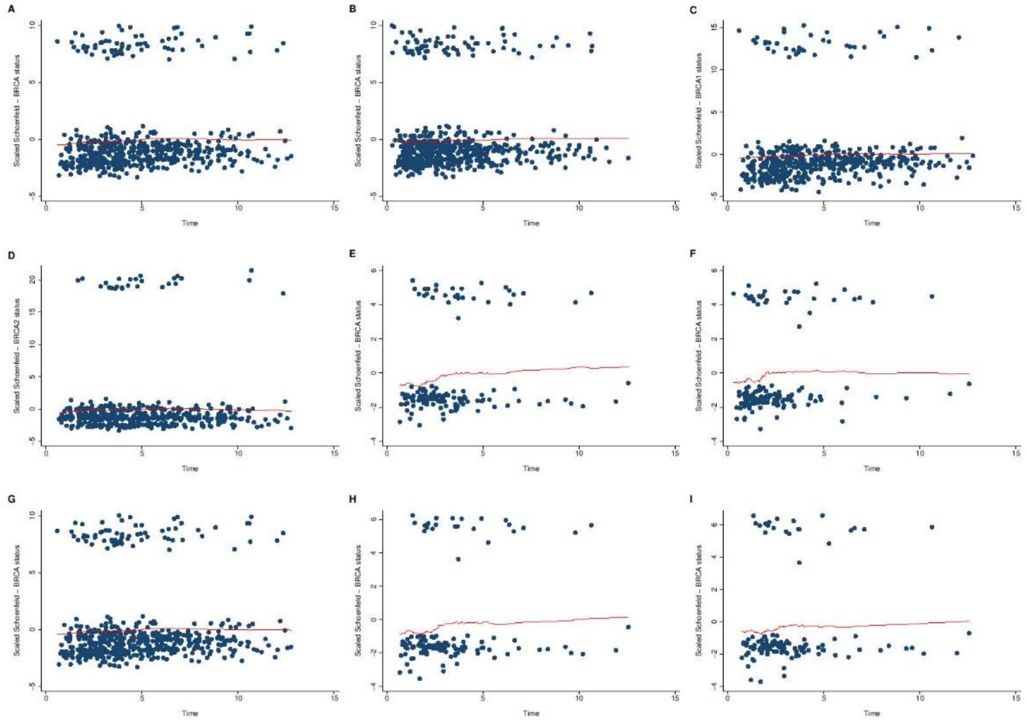

Appendix Figure 8 – Overall Survival by *BRCA* status for TNBC patients not having immediate bilateral mastectomies (TNBC population, excluding patients not having immediate bilateral mastectomies)

Forest Plot of univariable and multivariable hazard ratios by *BRCA*+/- status for Overall Survival (OS). Multivariable analysis is adjusted for age, body mass index, tumour size, ethnicity and use of taxane chemotherapy.

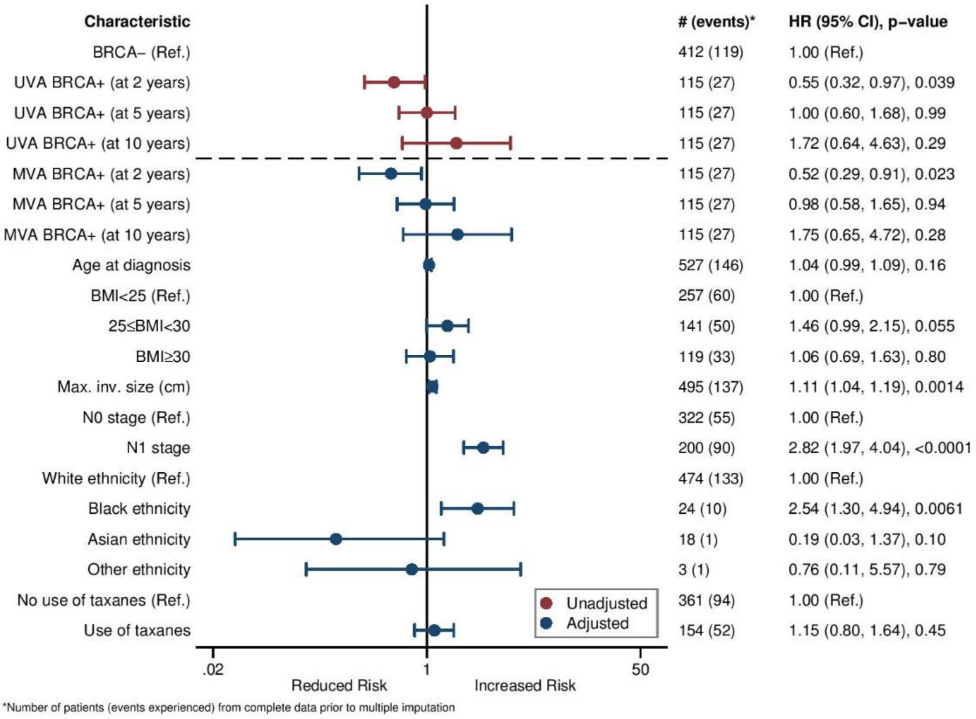

Appendix Figure 9 – Overall Survival by *BRCA* status for TNBC patients who did not develop a new primary breast or ovarian cancer (TNBC population, excluding patients who developed a new primary breast or ovarian cancer)
Forest Plot of univariable and multivariable hazard ratios by *BRCA*+/- status for Overall Survival (OS). Multivariable analysis is adjusted for age, body mass index, tumour size, ethnicity and use of taxane chemotherapy.

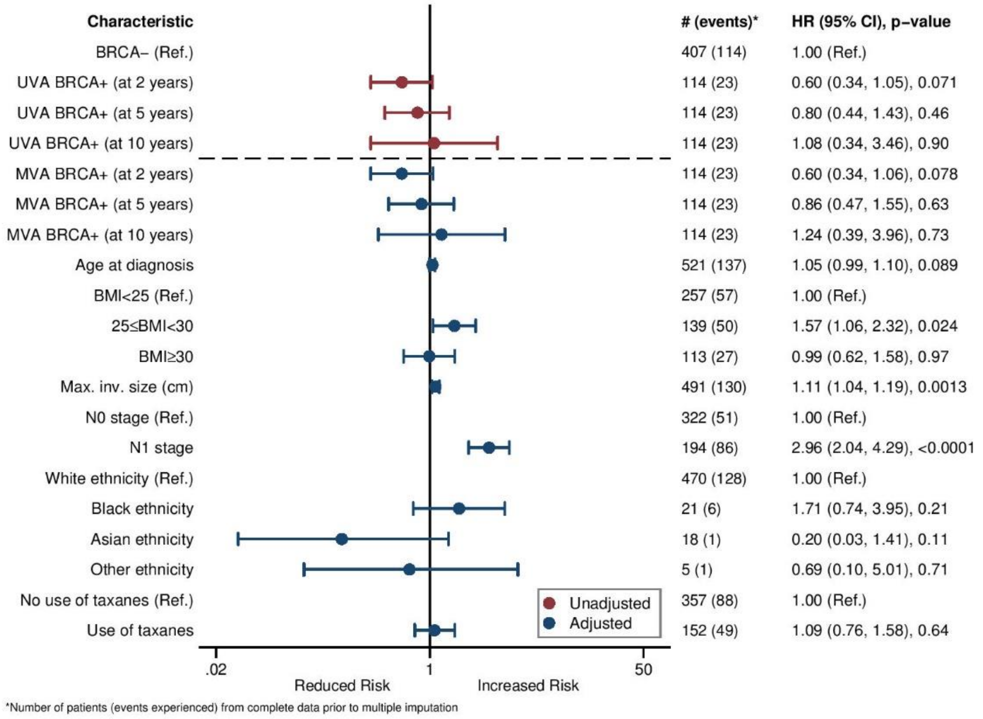

Appendix - Methods

Appendix Methods 1: BRCA1 and BRCA2 gene sequencing and variant calling
Details of sequencing methodology and annotation of variants.

Amplicon design, enrichment, sequencing, and variant calling:

All POSH study cases with a DNA sample submitted were included. Fluidigm targeted DNA amplification assay design software (Fluidigm, South San Francisco, California, USA) was used to select PCR ≤235bp amplicons covering all exons, splice junctions and UTRs of the BRCA1 and BRCA2 genes. These 261 amplicons were part of a larger multiplex panel of 1,122 amplicons covering 35 genes (manuscript in preparation). Using the Fluidigm software, primer pairs were multiplexed into 20 pools. The Fluidigm Juno Access Array 192.24 system was used for library preparation, according to the manufacturer's protocols (Fluidigm, South San Francisco, California, USA). Target sequences were amplified, then one of 1,536 unique sample barcodes and Illumina sequencing adaptors were ligated (supplied by Fluidigm, South San Francisco, California, USA). Liquid handling robotics and barcode plate identification were used in all steps of the library preparation process. Each library of 1,536 samples was quantified with the KAPA Library Quantification Kit (KapaBiosystems, Boston, Massachusetts, USA) and then sequenced in 150-base paired-end mode on a single lane of an Illumina Hi-Seq2000 instrument using v4 chemistry, according to the manufacturer's protocols (Illumina, San Diego, California, USA).

Raw sequence data were converted to FASTQ format and demultiplexed using the Illumina CASAVA v1.8 pipeline (Illumina, San Diego, California, USA. CutAdapt v1.5[1] was used for orientation-specific, end-wise primer sequence trimming, and untrimmed reads were discarded. Reads were aligned to the hg19 human reference sequence with BWA-MEM v0.7.[2]. Both SAMtools and GATK v3.3[3] was used for local insertion-deletion variant (indel) realignment and base quality score recalibration. Using intervals containing one or more full exons, GATK UnifiedGenotyper was used to perform SNP and indel discovery and variant calling across all samples simultaneously, according to the GATK best practice recommendations [4, 5]. We also called variants using a case by case approach which gave improved sensitivity and reduced specificity.

Sample and variant quality control (QC) filtering:

VCFtools[6] was used to first remove all variants with >20% missing calls, and then all samples with missing data for >20% of remaining variants. GATK was used to recalculate variant-level quality metrics for only the retained samples, and variant positions with quality by depth <3 or >25 were excluded. Genotypes with depth <20 or genotype quality <13 were recoded as no call using VCFtools. Finally, samples and then variants with >5% missing calls were excluded. After all filtering, 5,488/5,952 controls (92%) and 13,087/13,824 cases (95%) were retained for further analysis.
Indels with more than three alleles were removed. Potentially problematic variants, including indels longer than 1-bp in length, indels within 10-bp of one another, dinucleotide substitutions, and rare variants (defined by carrier frequency <0.1% in the ExAC Non-Finnish European dataset) for which one or more samples was called homozygous, were inspected manually in the Integrative Genome Viewer (IGV).[7] Where there were discrepancies between UnifiedGenotyper calls and the IGV inspection, the IGV-based variant call was used.

Functional prediction and variant frequency classification:

The Ensembl Variant Effect Predictor (VEP)[8] was used to assign the canonical transcript- and protein-level consequence for each variant. Frameshift, stop/gain, and canonical splice variants (i.e. positions -1,-2, +1 or +2) were considered as protein truncating. Missense variants were further annotated with effect predictions from CADD,[9] PolyPhen2,[10] SIFT,[11] and AlignGVGD,[12] a cancer gene-specific missense variant effect prediction tool. The consequences of the putative splice site variant CHEK2 c.320-5T>A were evaluated using the in silico prediction tools SpliceSiteFinder-like,[13] MaxEntScan,[14] NNSPLICE,[15] GeneSplicer,[16] and Human Splicing Finder.[17]

Coverage, quality, and variant call concordance metrics:

Per-sample and per-base mean sequence coverage were tabulated with BEDTools.[19]. For each sample, the GATK "callable loci" script was used to calculate the percentage of exonic bases with at least 20 reads and a minimum base quality of 20.
The accuracy of variant calling was assessed by Sanger sequencing to estimate the false positive rate (positive predictive value, PPV). Sanger sequencing primers with M13 sequence tags were designed. Sanger calls were checked against NGS results, and discrepancies were resolved via comparison of results and inspection of reads in IGV. Genotypes were successfully validated for 188/188 samples carrying SNVs (positive predictive value=100.0%) and 67/68 samples carrying indels (positive predictive value=98.5%).

Appendix Methods 1 – References:

1 Martin M. Cutadapt removes adapter sequences from high-throughput sequencing reads. EMBnet.journal 2011;17:10.

2 Li H. Exploring single-sample SNP and INDEL calling with whole-genome de novo assembly. Bioinformatics 2012;28:1838–44.
3 McKenna A, Hanna M, Banks E, Sivachenko A, Cibulskis K, Kernytsky A, *et al*. The Genome Analysis Toolkit: a MapReduce framework for analyzing next-generation DNA sequencing data. Genome Res 2010;20:1297–303.
4 DePristo MA, Banks E, Poplin R, Garimella K V, Maguire JR, Hartl C, *et al*. A framework for variation discovery and genotyping using next-generation DNA sequencing data. Nat Genet 2011;43:491–8.
5 Van der Auwera GA, Carneiro MO, Hartl C, Poplin R, del Angel G, Levy-Moonshine A, *et al*. From FastQ Data to High-Confidence Variant Calls: The Genome Analysis Toolkit Best Practices Pipeline. In: Current Protocols in Bioinformatics. John Wiley & Sons, Inc. 2013. 43:11.10.1-11.10.33.
6 Danecek P, Auton A, Abecasis G, Albers CA, Banks E, DePristo MA, Handsaker RE, Lunter G, Marth GT, Sherry ST, McVean G, Durbin R, 1000 Genomes Project Analysis Group. The variant call format and VCFtools. Bioinformatics 2011;27:2156–8.
7 Robinson JT, Thorvaldsdóttir H, Winckler W, Guttman M, Lander ES, Getz G, Mesirov JP. Integrative genomics viewer. Nat Biotechnol 2011;29:24–6.
8 McLaren W, Pritchard B, Rios D, Chen Y, Flicek P, Cunningham F. Deriving the consequences of genomic variants with the Ensembl API and SNP Effect Predictor. Bioinformatics 2010;26:2069–70.
9 Kircher M, Witten DM, Jain P, O'Roak BJ, Cooper GM, Shendure J. A general framework for estimating the relative pathogenicity of human genetic variants. Nat Genet 2014;46:310–5.
10 Adzhubei IA, Schmidt S, Peshkin L, Ramensky VE, Gerasimova A, Bork P, Kondrashov AS, Sunyaev SR. A method and server for predicting damaging missense mutations. Nat Methods 2010;7:248–9.
11 Ng PC, Henikoff S. Predicting deleterious amino acid substitutions. Genome Res 2001;11:863–74.
12 Tavtigian S V, Deffenbaugh AM, Yin L, Judkins T, Scholl T, Samollow PB, de Silva D, Zharkikh A, Thomas A. Comprehensive statistical study of 452 BRCA1 missense substitutions with classification of eight recurrent substitutions as neutral. J Med Genet 2006;43:295–305.
13 Zhang MQ. Statistical features of human exons and their flanking regions. Hum Mol Genet 1998;7:919–32.
14 Yeo G, Burge CB. Maximum entropy modeling of short sequence motifs with applications to RNA splicing signals. J Comput Biol 2004;11:377–94.
15 Reese MG, Eeckman FH, Kulp D, Haussler D. Improved Splice Site Detection in Genie. J Comput Biol 1997;4:311–23.
16 Pertea M, Lin X, Salzberg SL. GeneSplicer: a new computational method for splice site prediction. Nucleic Acids Res 2001;29:1185–90.
17 Desmet F-O, Hamroun D, Lalande M, Collod-Béroud G, Claustres M, Béroud C. Human Splicing Finder: an online bioinformatics tool to predict splicing signals. Nucleic Acids Res 2009;37:e67.
18 Lek M, Karczewski KJ, Minikel E V, Samocha KE, Banks E, Fennell T, *et al*. Analysis of protein-coding genetic variation in 60,706 humans. Nature 2016;536:285–91.
19 Quinlan AR, Hall IM. BEDTools: a flexible suite of utilities for comparing genomic features. Bioinformatics 2010;26:841–2.
20 Michailidou K, Hall P, Gonzalez-Neira A, Ghoussaini M, Dennis J, Milne RL, *et al*. Large-scale genotyping identifies 41 new loci associated with breast cancer risk. Nat Genet 2013;45:353–61.
21 Amos CI, Dennis J, Wang Z, Byun J, Schumacher FR, Gayther SA, *et al*: a Network for Understanding the Genetic Architecture of Common Cancers. Cancer Epidemiol Biomarkers Prev Published Online First: 3 October 2016. doi:10.1158/1055-9965.EPI-16-0106

Appendix - Documents

Appendix Document 1: Statistical Analysis Plan

Statistical analysis plan (SAP), approved on 10-May-2016, and formatted for Lancet Oncology Appendix.

[Please note: Figures in this SAP are taken from the POSH data available up until June 2015, and thus only represent approximations of the new data due to be downloaded from the POSH database in 2016/2017.]

Please note: This statistical analysis plan has been written in the past tense because it will form the basis of a paper. The headings used in this document come from the STROBE reporting guideline for observational studies (see http://www.strobe-statement.org/ or http://www.annals.org/content/147/8/W-163.full.pdf+html).

Statistical Analysis Plan Version

Issue no	Revision History	Author	Date
0.1	First draft written based on discussion at meeting on 8th Oct 2010	Louise Stanton (née Dent)	20th Oct 2010
0.2	Additional comments and annotations	Diana Eccles, Sue Gerty	13th Oct 2010
0.3	Further notes on confounding factors and example figures for POSH cohort added	Diana Eccles	25th Nov 2010
0.4	Updated based on meeting with Diana Eccles and Sue Gerty on the 29th Oct 2010 and meeting with Sue Gerty on 9th December 2010	Louise Stanton (née Dent)	17th Dec 2010
0.5	Updated based on comments from Doug Altman	Louise Stanton (née Dent)	21st Feb 2011
0.6	Updated based on discussions	Diana Eccles, Louise Stanton (née Dent)	24th Feb 2011
0.7	Updated based on meeting with Louise Stanton (née Dent) on 21st March 2012	Tom Maishman	30th Mar 2012
0.8	Updated based on comments from Diana Eccles	Tom Maishman	2nd Apr 2012
0.9	Updated following a meeting with Doug Altman, Diana Eccles and Louise Stanton (née Dent)	Tom Maishman	18th Mar 2013
0.10	Updated following planned updates to obtain further BRCA testing information	Tom Maishman	30th Jun 2015
0.11	Updated following comments from Diana Eccles and Ellen Copson	Tom Maishman	14th Jul 2015
0.12	Updated following comments from Diana Eccles and Ellen Copson	Tom Maishman	28th Jul 2015
0.13	Updated following meeting with Doug Altman on 30th July 2015	Tom Maishman	7th Aug 2015
1	Finalised using v0.13	Tom Maishman	10th May 2016

1. Introduction

1.1 Background / Rationale

BRCA1 and BRCA2 are the most frequently reported highly penetrant monogenic factors that predispose to breast cancer. Both genes also predispose to ovarian cancer. Mutation in either gene has been shown to lead to higher grade breast cancer than average and to young age at onset (median age for BRCA1 is 43 years and for BRCA2 is 48 years compared to the population mean age at diagnosis of about 60 years). In addition for BRCA1 associated breast cancer, the proportion of oestrogen receptor negative cancers is much higher than average (80-90% compared to ~ 30% amongst breast cancers in women diagnosed < 50 years of age). There are conflicting conclusions in the literature exploring whether BRCA1 or BRCA2 mutation carriers develop breast cancers with a better or worse prognosis. Most reported studies are small, retrospective and with incomplete data on many of the factors known to influence breast cancer outcomes. Some of the early reports of better survival failed to recognise or adequately account for survival bias in many of the BRCA tested patients. Knowledge of a family history of breast cancer, even without genetic testing may lead to earlier diagnosis of breast cancer due to heightened awareness and early presentation and investigation; this bias may lead to observations of improved survival in BRCA gene carriers. The adverse pathological features associated with breast cancers diagnosed in BRCA gene carriers may account for observations of a worsened prognosis in gene carriers compared with the average.. A differentially better or worse response to adjuvant chemotherapy in relation to the underlying genetic predisposition may also affect prognosis. It is important to understand the overall effect of genetic predisposition factors on prognosis in order to better inform gene carriers making decisions about primary prevention and about cancer treatment and to help design more informative prospective clinical trials of both conventional and novel targeted treatments. The Prospective study of Outcomes in Sporadic versus Hereditary breast cancer (POSH) is a large contemporary cohort study of breast cancer cases diagnosed before 41 years of age and designed to investigate the effect of genetic factors on breast cancer prognosis.

1.2 Objectives

This paper presents the results from analyses carried out on data collected from the POSH study.

The primary objective was:

- To investigate whether patients with early breast cancer and an inherited BRCA1 or BRCA2 gene mutation (BRCA-Positive [BRCA+]) have a superior Overall Survival (OS) than patients without a BRCA1 or BRCA 2 mutation (BRCA-Negative [BRCA-]).

Secondary objectives were:

- To investigate whether BRCA+ patients with early breast cancer have a superior Distant Disease Free Survival (DDFS) than BRCA- patients.
- To investigate whether BRCA+ patients with early breast cancer have a superior Post Distant Relapse Survival (PDRS) than BRCA- patients.

- To investigate whether patients with early breast cancer and an inherited BRCA1 gene mutation (BRCA1-Positive [BRCA1+]) have a superior OS than patients without a BRCA1 mutation (BRCA1-Negative [BRCA1-])[1].
- To investigate whether BRCA1+ patients with early breast cancer have a superior DDFS than BRCA1- patients.
- To investigate whether BRCA1+ patients with early breast cancer have a superior PDRS than BRCA1- patients.
- To investigate whether patients with early breast cancer and an inherited BRCA2 gene mutation (BRCA2-Positive [BRCA2+]) have a superior OS than patients without a BRCA2 mutation (BRCA2-Negative [BRCA2-])[2].
- To investigate whether BRCA2+ patients with early breast cancer have a superior DDFS than BRCA2- patients.
- To investigate whether BRCA2+ patients with early breast cancer have a superior PDRS than BRCA2- patients.

- To investigate whether Triple Negative (TNT)[3] BRCA+ patients with early breast cancer have a superior OS than TNT BRCA- patients.
- To investigate whether TNT BRCA+ patients with early breast cancer have a superior DDFS than TNT BRCA- patients.
- To investigate whether TNT BRCA+ patients with early breast cancer have a superior PDRS than TNT BRCA- patients.

- To investigate whether BRCA+ patients with early breast cancer have a superior DDFS than BRCA- patients when adjusting for chemotherapy.

[1] This comparison excludes patients with a BRCA2 positive gene mutation.
[2] This comparison excludes patients with a BRCA1 positive gene mutation.
[3] Triple Negative Patients defined as Patients with a HER2 negative status, ER negative status and either a PR negative status or PR missing/unknown status i.e. patients with a confirmed PR positive status are excluded.

2. Methods

2.1 Study Design
The POSH study is a prospective cohort study. The protocol for the study can be found in the following journal article http://www.biomedcentral.com/1471-2407/7/160.

2.2 Setting
The POSH study recruited women from breast cancer units across England, Scotland, Wales and Northern Island between 1st June 2001 to 31st January 2008.

2.3 Participants
The study recruited 3052 women aged 40 years or younger at breast cancer diagnosis. The women had to have been diagnosed with breast cancer between January 2000 and January 2008. In addition, 43 women aged 41-50 were also included if they had a known BRCA1 or BRCA2 gene mutation and were diagnosed with invasive breast cancer within the study period were excluded for this analysis. Women were excluded if they had a previous invasive malignancy (with the exception of non-melanomatous skin cancer), were not available for follow up or refused consent to retain diagnostic and follow up data. Genetic testing was performed on xxx women. Those not tested were excluded from the additional comparison. Patients with confirmed M1 stage (n=74) were also excluded. A total of 2925 women were included in the analysis population.

Clinical follow up data were obtained from the patient medical records by the clinical trials practitioner (CTP) at each recruiting centre. Data forms collecting information at diagnosis, 6 months, 12 months were completed by the CTP usually at 12 months from diagnosis. Annual data collection was continued from the date of definitive diagnosis until death, loss to follow up or until the end of the current phase of the study (mmm yyyy).

Family history data: patients in the POSH study completed a family history questionnaire (http://www.biomedcentral.com/1471-2407/7/160 supplementary figure). The web-based and validated genetic risk prediction software BOADICEA (Antoniou A, et al 2008. Predicting the likelihood of carrying a BRCA1 or BRCA2 mutation: validation of BOADICEA, BRCAPRO, IBIS, Myriad and the Manchester scoring system using data from UK genetics clinics. J Med Genet. Jul;45(7):425-31) was used to process pedigree data and generate a predicted likelihood that each patient might carry a BRCA1/2 mutation. No family history was provided for 106 of the 2956 patients. BOADICEA scores for the remaining 2850 patients were calculated from the family history of the proband at the time she presented with breast cancer. A total of 1939 (66%) scored below 0.05, 372 (13%) scored 0.05-0.099, 226 (8%) scored 0.10-0.199 and 314 (11%) scored 0.20 or over. BOADICEA scores for the xxx patients were calculated from the family history of the proband at the time she presented with breast cancer.
Genetic testing results for BRCA1/2 were already available through clinical test reports or other research sub-studies in xxx cases and these data were used to validate the sensitivity and specificity of the Fluidigm technology used across the cohort. Mutation testing was carried out on all patients recruited to the study for whom a DNA sample was available (n=xxx). A panel of genes was tested using Fluidigm targeted sequence capture and next generation sequencing with additional analysis using Multiple Ligation Probe Analysis (MLPA) to detect large exonic deletions or duplications where there was either a greater than 10% estimated probability of an underlying BRCA1/2 gene mutation (estimated using BOADICEA) or where there was evidence from the Fluidigm assay of a large deletion or duplication. Only mutations that were clearly pathogenic were used to assign gene carriers to the relevant group for analysis purposes.

2.4 Variables (data taken as of June 2015)

Variable	Type of data / categories	Amount of missing data (Analysis Group A – see Section 2.8, n=2873)	Amount of missing data (Analysis Group B – see Section 2.8, n=725)	Possible reasons for missing data
**2.4.1 Primary outcome**				
Time to death from any cause	Survival data<br><br>Date of death from any cause – Date of invasive breast cancer diagnosis	N/A, patients who haven't died will be censored at the date of their last follow up visit	N/A, patients who haven't died will be censored at the date of their last follow up visit	N/A
**2.4.2 Secondary outcomes**				
Time to distant relapse or death from any cause	Survival data<br><br>Date of first distant relapse (or death from any cause) – Date of invasive breast cancer diagnosis	N/A, patients who haven't relapsed or died will be censored at the date of their last follow up visit	N/A, patients who haven't relapsed or died will be censored at the date of their last follow up visit	N/A
Time from first relapse to death from any cause	Survival data<br><br>Date of death from any cause – Date of first distant relapse	N/A, patients who haven't relapsed will not be included. Patients who have relapsed and haven't died will be censored at the date of their last follow up visit	N/A, patients who haven't relapsed will not be included. Patients who have relapsed and haven't died will be censored at the date of their last follow up visit	N/A
**2.4.3 Candidate predictor**				
Genetic status[1]	Categorical<br>For the main comparison, each patient is assigned one of 3 categories:<br>BRCA 1 gene carrier confirmed by genetic testing (n=xxx)<br>BRCA 2 gene carrier confirmed by genetic testing (n=xxx)<br>TP53 (n=xxx)<br>No mutation found/variant unknown significance	TBA	TBA	TBA
**2.4.4 Potential confounders / effect modifiers - measured at breast cancer diagnosis presentation**				
1. Age at diagnosis	Continuous, in years	0 records	0 records	N/A
2. Body Mass Index (BMI)	Categorical<br>Underweight/Healthy, Overweight, Obese, or missing/unknown	108 (3.8%) records	15 (2.1%) records	Consider MAR
3. Histological Tumour grade	Categorical<br>1, 2, 3, or not graded/missing/unknown	70 (2.4%) records not graded/missing/unknown	19 (2.6%) records not graded/missing/unknown	MCAR. Inadequate reporting by pathologist. If grade of core biopsy tumour not stated, and after neo-adjuvant chemotherapy there was a complete pathological response then no tumour to report on.
4. Maximum tumour diameter invasive (tumour size)	Continuous, in mm<br>or<br>Categorical<br><15mm, 15mm to 20mm, >20mm to 35mm, >35mm to	162 (5.6%) records	53 (7.3%) records	Missing for similar reasons as tumour grade (MCAR)

Variable	Type of data / categories	Amount of missing data (Analysis Group A – see Section 2.8, n=2873)	Amount of missing data (Analysis Group B – see Section 2.8, n=725)	Possible reasons for missing data
	50mm, >50mm, or missing/unknown			
5. Pathological N stage (lymph node status)	Categorical N0, N1 or missing/unknown	31 (1.1%) records	10 (1.4%) records	MCAR. No axillary surgery, no lymph nodes in resected specimen.
6. Number of positive Lymph nodes	Categorical 0, 1-3, 4-9, 10+, or missing/unknown	31 (1.1%) records	10 (1.4%) records	Same as above (MCAR)
7. Lymphovascular invasion	Categorical Present, absent or missing/unknown	203 (7.1%) records	58 (8.0%) records	Poor reporting. Consider as MCAR.
8. M stage	Categorical M0, M1 or missing/unknown	22 (0.8%) records	5 (0.7%) records	MCAR, likely to be M0 as only 2.1% of patients are M1.
9. Oestrogen receptor (ER)[1]	Categorical Negative, positive, or missing/unknown	11 (0.4%) records	0 records	N/A
10. HER2[2]	Categorical Negative, positive, or missing/unknown	352 (12.3%) records	0 records	Missing because diagnosis predated routine testing and patient has not suffered a further breast cancer event since initial diagnosis. Consider Missing At Random (MAR).
11. PR[3]	Categorical Negative, positive, or missing/unknown	564 (19.6%) records	85 (11.7%) records	MAR. Missing because specific centres don't do PR IHC.
12. Ethnicity	Categorical Caucasian/White, Black, Asian, Other, or missing/unknown	41 (1.4%) records	8 (1.1%) records	Consider MAR
Diagnosis Year	Categorical ≤2005 or >2005	0 records	0 records	N/A
Adjuvant or neo-adjuvant chemotherapy indicator	Categorical Yes or No/missing/unknown	0 records	0 records	N/A
Chemotherapy with taxane indicator	Categorical Yes or No/missing/unknown	0 records	0 records	N/A
17. Focality (distribution of tumour)	Categorical Multifocal, localised or missing/unknown	61 (8.0%) records	286 (9.7%) records	Missing for similar reasons as tumour grade (MCAR).
18. Definitive surgery	Categorical Breast Conserving Surgery (BCS), Mastectomy, No surgery, Nodal surgery only, or missing/unknown	0 records	0 records	N/A
19. Chemotherapy regimen	Categorical Anthracyclines, A&T, Taxanes, Other, or None	0 records	0 records	N/A
**2.4.5 Additional (descriptive) variables**				
13. Length of follow-up	Continuous, in months	0 records	0 records	N/A
**Amount of missingness in the multivariable models**				
**No. of pts with at least 1 variable with missing data from the MV model 1 (see Section 2.8)**		**596 (20.7%)**	**155 (21.4%)**	
**No. of pts with at least 1 variable with missing data from the MV model 2 (see Section 2.8)**		**610 (21.2%)**	**159 (21.9%)**	

[1] Not all patients in the POSH study had genetic testing (in the same way not all patients do currently in the NHS). BOADICEA scores were calculated purely based on family history data from the patient family history questionnaire; no information about mutation testing was included in the estimates. Patients with a combined (BRCA1 and BRCA2) score of <0.05 had no significant family history of cancer. Scores above 0.10 would be eligible for testing according to American Society of Oncology guidelines and scores above 0.10 are eligible for testing under the 2013 UK NICE guidelines.

[2] Oestrogen receptor allocation of result from POSH database to Oestrogen receptor category:

Result	Category result assigned to
Negative	Negative
Borderline	Negative
Strongly Positive	Positive
Positive	Positive
Weakly positive	Negative*
Not done	Not done
Unknown	Missing/unknown
Null	Missing/unknown

*For ER, weakly positive (which we assume equates to an Allred score of 1-2) has been treated as ER negative, and an Allred socre of 3+ treated as ER positive. However it is possible that reviewers will disagree so we can reclassify this as positive if required.

[3] HER2 allocation of result from POSH database to a HER2 category:

Result	Category result assigned to
FISH/CISH positive	Positive**
3+	Positive
FISH/CISH borderline	Negative**
2+	Negative
FISH/CISH negative	Negative**
1+	Negative
0	Negative
Not done	Not done
Unknown	Missing/unknown

** FISH/CISH results take precedence i.e. a 2+ result which is later found to have a FISH/CISH positive result is categorised as Positive rather than Borderline.

[4] PR allocation of result from POSH database to a PR category:

Result	Category result assigned to
Negative	Negative
Borderline	Negative
Strongly Positive	Positive
Positive	Positive
Weakly positive	Negative***
Not done	Not done
Unknown	Missing/unknown
Null	Missing/unknown

***For PR, weakly positive (which we assume equates to an Allred score of 1-2) has been treated as PR negative. However it is possible that reviewers will disagree so we can reclassify this as positive if required

2.5 Data sources/measurement

The tumour biopsy, definitive histopathological report, clinical and radiological reports were all submitted to the study. Pathological characteristics of the tumours were taken from the diagnostic histopathology report, clinical staging from the clinical and radiological reports.

National death data were obtained for patients in the cohort from the Medical Research Information Service (MRIS).

ER, PR and HER2 data were taken from pathology reports. Scoring systems varied as expected across contributing hospitals. Positive and Negative categories are straightforward however borderline results exist in all three IHC categories and were classified into a separate borderline group. The borderline category was merged with negative for the purposes of these analyses. Additional IHC data for these three markers was available from the Tissue Micro Arrays (TMAs) constructed from tumour pathology blocks for study participants which were used to populate these missing clinical data fields.

This paper presents the results of analyses conducted on follow up data available up until dd-mmm-yyyy.

2.6 Bias

Clinical data for all patients were collected via standard clinical research forms which were completed from the clinical notes by the Clinical Trials Practitioner in each centre.

HER2 data: There are concerns regarding the amount of missing HER2 data obtained. In addition:

- HER2 Testing was only widely introduced after 2006 (proportion tested prior to 2006 was 83% (1704/2041), proportion tested on/after 2006 was 98% (897/915)). Prior to 2006 HER2 testing was more likely to have been carried out in patients who had progressed (93% i.e. 520 tested out of 561 who progressed, compared to 80% i.e. 1184 tested out of 1480 who had not progressed). Therefore, patients for whom we knew their HER2 status were more likely to have had a worse prognosis. Hence, if we selected patients on the basis of HER2 testing and compared them to patients who may or may not have been HER2 tested this would have been biased as the patients who have been HER2 tested could look worse by comparison.

- In addition, any analyses that select any patients which have a known HER2 status (which includes patients diagnosed before 2006) will include more cases who had relapsed (and were therefore tested for HER2 amplification retrospectively) than the whole cohort which could potentially compromise the validity of results.

2.7 Study Size

This is covered in the BMC paper.

2.8 Statistical Methods

Patients excluded from the analyses
Patients were excluded from this analysis if we didn't have confirmation that they had invasive cancer from pathology results or were missing primary data (21 patients). Genetic testing was performed on xxx women. Those not tested were excluded from the additional comparison. Patients with confirmed M1 stage (n=74) were also excluded. A total of 2925 women were included in the analysis population, of which:

- n=2873 were aged 40 years or younger at diagnosis without a TP53 gene mutation **(Analysis Group A)**;
- n=725 were aged 40 years or younger at diagnosis without a TP53 gene mutation and had a TNT status **(Analysis Group B)**;
- n=43 were aged 41-50 years at diagnosis with a confirmed gene mutation **(Analysis Group C)**;
- n=9 were aged 40 years or younger at diagnosis and had a TP53 gene mutation **(Analysis Group D)**.

Primary outcome measure
Overall Survival (OS) where OS is defined as the time from the date of invasive breast cancer diagnosis to death from any cause. Patients who had not died will be censored at their date of last follow up. For this analysis we will not include new primary breast cancer diagnoses as distant relapse events in the primary outcome analysis.

Secondary outcome measures
Distant Disease Free Survival (DDFS) where DDFS is defined as the time from the date of invasive breast cancer diagnosis to distant relapse or death from any cause. Distant relapse is defined as breast cancer recurrence at distant sites including supraclavicular lymph nodes, visceral, CNS and bone metastases. Patients who had not died or relapsed at the time of analysis will be censored at their date of last follow up. For this analysis we will not include new primary breast cancer diagnoses as distant relapse events in the secondary outcome analysis.

Post Distant Relapse Survival (PDRS) where PDRS is defined as the time from the date of distant relapse to death from any cause. Distant relapse is defined as breast cancer recurrence at distant sites including supraclavicular lymph nodes, visceral, CNS and bone metastases. Patients who had not died will be censored at their date of last follow up. For this analysis we will not include new primary breast cancer diagnoses as distant relapse events in the secondary outcome analysis.

Univariate analyses
Where specified for analysis groups A, B, C and D above, we summarised patient and tumour characteristics by the following:

- All patients (Analysis **Groups A, B, C and D**)
- BRCA1+ patients (Analysis **Groups A, B and C** only)
- BRCA2+ patients (Analysis **Groups A, B and C** only)
- BRCA+ patients (Analysis **Groups A and B** only)
- BRCA- patients (Analysis **Groups A and B** only)

For analysis groups A and B, we summarised and produced Kaplan Meier survival curves of OS, DDFS, and PDRS and compared the survival curves using a log rank test for the following:

- BRCA+ versus BRCA-
- BRCA1+ versus BRCA1- (excluding BRCA2+ patients)
- BRCA2+ versus BRCA2- (excluding BRCA1+ patients)

For analysis group C, we summarised and produced Kaplan Meier survival curves of OS, DDFS, and PDRS and compared the survival curves using a log rank test for BRCA1+ versus BRCA2+patients.

Multivariable analyses
Comparison groups:

- BRCA+ versus BRCA- **(analysis Group A)**
- BRCA1+ versus BRCA1-(excluding BRCA2+ patients) **(analysis Group A)**
- BRCA2+ versus BRCA2- (excluding BRCA1+ patients) **(analysis Group A)**
- TNT BRCA+ versus TNT BRCA- **(analysis Group B)**

For the comparisons i) to iv) above, we fitted a multivariable model for OS and DDFS adjusting for the following covariates:

- Age at diagnosis, in years (fitted as a continuous covariate);
- Body Mass Index (BMI) (fitted as a categorical covariate [Underweight/Healthy, Overweight or Obese]);
- Histological Grade (fitted as a categorical covariate [1, 2 or 3]);
- Maximum invasive tumour size, in mm (fitted as a continuous covariate);
- N stage (fitted as a binary covariate [N0 or N1]);
- ER status (fitted as a binary covariate [Negative or Positive]) **(for analysis Group A only)**;
- HER2 status (fitted as a binary covariate [Negative or Positive]) **(for analysis Group A only)**;

For the comparisons i) to iv) above, we fitted a multivariable model for OS and DDFS, comparing BRCA+ versus BRCA-, adjusting for the following covariates:

- Age at diagnosis, in years (fitted as a continuous covariate);
- Body Mass Index (fitted as a categorical covariate [Underweight/Healthy, Overweight or Obese]);
- Histological Grade (fitted as a categorical covariate [1, 2 or 3]);
- Maximum invasive tumour size, in mm (fitted as a continuous covariate);
- N stage (fitted as a binary covariate [N0 or N1]);
- ER status (fitted as a binary covariate [Negative or Positive]) **(for analysis Group A only)**;
- HER2 status (fitted as a binary covariate [Negative or Positive]) **(for analysis Group A only)**;
- Ethnicity (fitted as a categorical covariate [Caucasian, Black or Asian]) – *where appropriate*;
- Diagnosis Year (fitted as a binary covariate [≤2005, or >2005]) – *where appropriate*;
- Adjuvant or neo-adjuvant chemotherapy indicator (fitted as a binary covariate [yes, or no/missing/unknown]) – *where appropriate*;
- Chemotherapy with taxane indicator (fitted as a binary covariate [yes-with taxane, or no-without taxane]) – *where appropriate*.

Hazard Ratios

Evidence suggests that the effect of ER status changes over time (Azzato, et al, 2009, Bellera et al, 2010)[1]. Indeed, this was evident after testing the proportional hazards assumption based on the Schoenfeld residuals and using the identity matrix for the time-scaling function[2] i.e. using the estat phtest command in STATA. This result provided strong evidence against the Cox proportional hazards assumption ($p<0.001$), which was also seen when plotting the scaled Schoenfeld residuals over time[2].

As a result of the time-varying effects of the ER status, a flexible parametric survival model was programmed in STATA using the stpm2 command (Lambert, Royston, 2009)[3] to model ER as a time-dependent covariate. The degrees of freedom for the restricted cubic spline function used for the hazard rate was set to the default setting of 3, whilst the degrees of freedom for the time-dependent effects was set so as to provide the lowest Akaike information criterion (AIC) and Bayesian information criterion (BIC). The time-varying hazard ratio and 95% confidence interval was plotted over time and 2-, 5-, and 8-year relative hazard ratios and survival estimates were produced.

[1]The Azzato, et al paper can be found at http://www.ncbi.nlm.nih.gov/pmc/articles/PMC2695697/. The Bellera et al paper can be found at http://www.biomedcentral.com/1471-2288/10/20.
[2]Results obtained from T Maishman's MSc Project analysis undertaken on POSH data downloaded in May 2011.
[3] The Lambert & Royston paper can be found at www.stata-journal.com/article.html?article=st0165 or http://www.pauldickman.com/cancerepi/handouts/handouts_survival/Lambert2009.pdf

Method used to handle missing data

The amount of missingess will be investigated and if deemed appropriate, methods of multiple imputation will be incorporated. Otherwise, a complete-case analysis approach will be incorporated.

To date, between 20-22% of patients have are missing data for at least 1 covariate in the multivariable models.

Appendix Document 2: STROBE Checklist
Strengthening the Reporting of Observational Studies in Epidemiology (STROBE) checklist.

	Item No.	Recommendation	Page No.	Relevant text from manuscript
**Title and abstract**	1	(*a*) Indicate the study's design with a commonly used term in the title or the abstract	1 (and 3)	Within the title (1) and abstract (3)
		(*b*) Provide in the abstract an informative and balanced summary of what was done and what was found	3	Within the abstract (Methods and Findings)
**Introduction**				
Background/rationale	2	Explain the scientific background and rationale for the investigation being reported	4-5	Within the Background
Objectives	3	State specific objectives, including any prespecified hypotheses	5	Within the Background
**Methods**				
Study design	4	Present key elements of study design early in the paper	5	Within the Background and Methods
Setting	5	Describe the setting, locations, and relevant dates, including periods of recruitment, exposure, follow-up, and data collection	5-7	Within the Methods
Participants	6	(*a*) *Cohort study*—Give the eligibility criteria, and the sources and methods of selection of participants. Describe methods of follow-up<br>*Case-control study*—Give the eligibility criteria, and the sources and methods of case ascertainment and control selection. Give the rationale for the choice of cases and controls<br>*Cross-sectional study*—Give the eligibility criteria, and the sources and methods of selection of participants	5-6	Within the Methods
		(*b*) *Cohort study*—For matched studies, give matching criteria and number of exposed and unexposed<br>*Case-control study*—For matched studies, give matching criteria and the number of controls per case	N/A	N/A
Variables	7	Clearly define all outcomes, exposures, predictors, potential confounders, and effect modifiers. Give diagnostic criteria, if applicable	5-8	Within the Methods
Data sources/ measurement	8*	For each variable of interest, give sources of data and details of methods of assessment (measurement). Describe comparability of assessment methods if there is more than one group	5-7	Within the Methods
Bias	9	Describe any efforts to address potential sources of bias	8	Within the Methods
Study size	10	Explain how the study size was arrived at	7	Within the Methods

Continued on next page

Quantitative variables	11	Explain how quantitative variables were handled in the analyses. If applicable, describe which groupings were chosen and why	7-8	Within the Methods
Statistical methods	12	(*a*) Describe all statistical methods, including those used to control for confounding	7-8	Within the Methods
		(*b*) Describe any methods used to examine subgroups and interactions	7-8	Within the Methods
		(*c*) Explain how missing data were addressed	8	Within the Methods
		(*d*) *Cohort study*—If applicable, explain how loss to follow-up was addressed<br>*Case-control study*—If applicable, explain how matching of cases and controls was addressed<br>*Cross-sectional study*—If applicable, describe analytical methods taking account of sampling strategy	8	Within the Methods
		(*e*) Describe any sensitivity analyses	8	Within the Methods
**Results**				
Participants	13*	(a) Report numbers of individuals at each stage of study—eg numbers potentially eligible, examined for eligibility, confirmed eligible, included in the study, completing follow-up, and analysed	8-9 & Appendix Figure 1	Within the Results & Appendix Figure 1
		(b) Give reasons for non-participation at each stage	8-9 & Appendix Figure 1	Within the Results & Appendix Figure 1
		(c) Consider use of a flow diagram	Appendix Figure 1	Within Appendix Figure 1
Descriptive data	14*	(a) Give characteristics of study participants (eg demographic, clinical, social) and information on exposures and potential confounders	8-9, Tables 1 & 2, Appendix Figure 1	Within the Results, Tables 1 & 2, & Appendix Figure 1
		(b) Indicate number of participants with missing data for each variable of interest	Tables 1 & 2	Within the Tables 1 & 2
		(c) *Cohort study*—Summarise follow-up time (eg, average and total amount)	9	Within the Results
Outcome data	15*	*Cohort study*—Report numbers of outcome events or summary measures over time	9-10, Figures 1 & 2, Appendix Figures 2, 3, 4, 5, 6, 8, & 9	Within the Results, Figures 1 & 2, & Appendix Figures 2, 3, 4, 5, 6, 8, & 9
		Case-control study—Report numbers in each exposure category, or summary measures of exposure	N/A	N/A
		Cross-sectional study—Report numbers of outcome events or summary measures	N/A	N/A
Main results	16	(*a*) Give unadjusted estimates and, if applicable, confounder-adjusted estimates and their precision (eg, 95% confidence interval). Make clear which confounders were adjusted for and why they were included	9-11, Figures 1 & 2, Appendix Figures 2, 3, 4, 5, 6, 8, & 9	Within the Results, Figures 1 & 2, & Appendix Figures 2, 3, 4, 5, 6, 8, & 9
		(*b*) Report category boundaries when continuous variables were categorized	Tables 1 & 2	Within Tables 1 & 2
		(*c*) If relevant, consider translating estimates of relative risk into absolute risk for a meaningful time period	N/A	N/A

Continued on next page

Other analyses	17	Report other analyses done—eg analyses of subgroups and interactions, and sensitivity analyses	10-11, Appendix Figures 8 & 9	Within the Results and Appendix Figures 8 & 9 for post-hoc analyses results
**Discussion**				
Key results	18	Summarise key results with reference to study objectives	11-13	Within the Discussion
Limitations	19	Discuss limitations of the study, taking into account sources of potential bias or imprecision. Discuss both direction and magnitude of any potential bias	14-15	Within the Discussion
Interpretation	20	Give a cautious overall interpretation of results considering objectives, limitations, multiplicity of analyses, results from similar studies, and other relevant evidence	15	Within the Discussion
Generalisability	21	Discuss the generalisability (external validity) of the study results	13-15	Within the Discussion
**Other information**				
Funding	22	Give the source of funding and the role of the funders for the present study and, if applicable, for the original study on which the present article is based	4, 8, 16	Within the Funding section following the abstract, within the Methods and within Acknowledgements

*Give information separately for cases and controls in case-control studies and, if applicable, for exposed and unexposed groups in cohort and cross-sectional studies.

Note: An Explanation and Elaboration article discusses each checklist item and gives methodological background and published examples of transparent reporting. The STROBE checklist is best used in conjunction with this article (freely available on the Web sites of PLoS Medicine at http://www.plosmedicine.org/, Annals of Internal Medicine at http://www.annals.org/, and Epidemiology at http://www.epidem.com/). Information on the STROBE Initiative is available at www.strobe-statement.org.

Appendix Figure 10 – Time-varying effects of BRCA status on Overall Survival for all TNBC patients (TNBC population)
Time-varying hazard rates by BRCA1 and/or 2 status (BRCA+/-) for Overall Survival (OS) (Panel A); and corresponding time-varying hazard ratio for Overall Survival (Panel B).

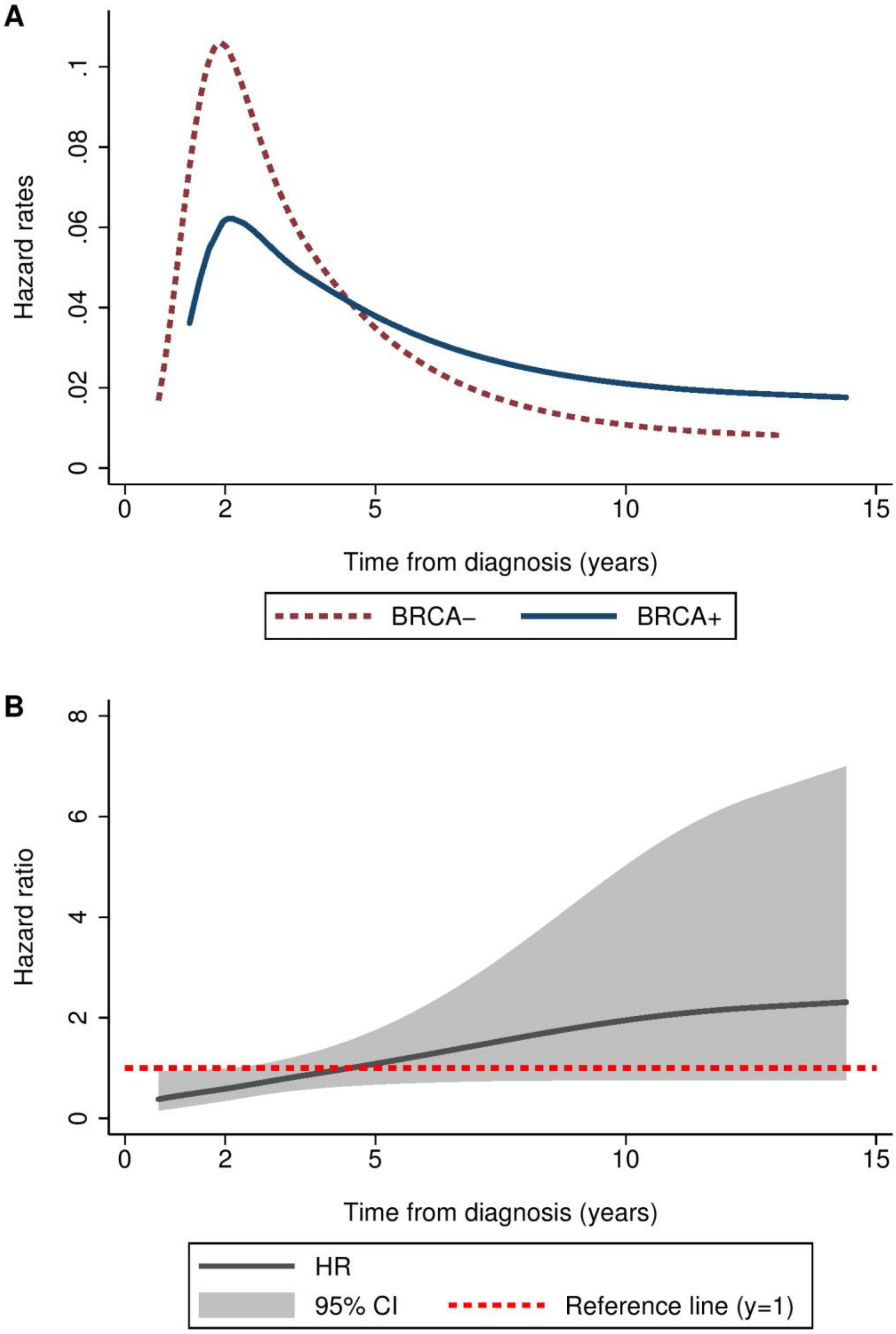

Supplement: Supplementary appendix [file mmc1.pdf]
